# Iterated Virtual Screening-Assisted Antiviral and Enzyme Inhibition Assays Reveal the Discovery of Novel Promising Anti-SARS-CoV-2 with Dual Activity

**DOI:** 10.3390/ijms22169057

**Published:** 2021-08-22

**Authors:** Rania Hamdy, Bahgat Fayed, Ahmed Mostafa, Noura M. Abo Shama, Sara Hussein Mahmoud, Chetan Hasmukh Mehta, Yogendra Nayak, Sameh S. M. Soliman

**Affiliations:** 1 Research Institute for Medical and Health Sciences, University of Sharjah, Sharjah 27272, United Arab Emirates; rania_hamdy2000@yahoo.com (R.H.); bfayed@sharjah.ac.ae (B.F.); 2 Faculty of Pharmacy, Zagazig University, Zagazig 44519, Egypt; 3 Chemistry of Natural and Microbial Product Department, National Research Centre, Cairo 12622, Egypt; 4 Centre of Scientific Excellence for Influenza Viruses, National Research Centre, Giza 12622, Egypt; ahmed_elsayed@daad-alumni.de (A.M.); noura.mahrous1995@gmail.com (N.M.A.S.); sarahussein9@yahoo.com (S.H.M.); 5 Manipal College of Pharmaceutical Sciences, Manipal Academy of Higher Education, Manipal 576104, India; chetan_jd12@rediffmail.com (C.H.M.); yogendra.nayak@manipal.edu (Y.N.); 6 College of Pharmacy, University of Sharjah, Sharjah 27272, United Arab Emirates

**Keywords:** COVID-19, SARS-CoV-2, iterated virtual screening, M^pro^, TMPRSS2, in vitro antiviral activity

## Abstract

Unfortunately, COVID-19 is still a threat to humankind and has a dramatic impact on human health, social life, the world economy, and food security. With the limited number of suggested therapies under clinical trials, the discovery of novel therapeutic agents is essential. Here, a previously identified anti-SARS-CoV-2 compound named **Compound 13** (1,2,5-Oxadiazole-3-carboximidic acid, 4,4′-(methylenediimino) bis,bis[[(2-hydroxyphenyl)methylene]hydrazide) was subjected to an iterated virtual screening against SARS-CoV-2 M^pro^ using a combination of Ligand Designer and PathFinder. PathFinder, a computational reaction enumeration tool, was used for the rapid generation of enumerated structures via default reaction library. Ligand designer was employed for the computerized lead optimization and selection of the best structural modification that resulted in a favorable ligand–protein complex. The obtained compounds that showed the best binding to M^pro^ were re-screened against TMPRSS2, leading to the identification of 20 shared compounds. The compounds were further visually inspected, which resulted in the identification of five shared compounds **M1–5** with dual binding affinity. In vitro evaluation and enzyme inhibition assay indicated that **M3**, an analogue of **Compound 13** afforded by replacing the phenolic moiety with pyridinyl, possesses an improved antiviral activity and safety. **M3** displayed in vitro antiviral activity with IC_50_ 0.016 µM and M^pro^ inhibition activity with IC_50_ 0.013 µM, 7-fold more potent than the parent **Compound 13** and potent than the antivirals drugs that are currently under clinical trials. Moreover, **M3** showed potent activity against human TMPRSS2 and furin enzymes with IC_50_ 0.05, and 0.08 µM, respectively. Molecular docking, WaterMap analysis, molecular dynamics simulation, and R-group analysis confirmed the superiority of the binding fit to **M3** with the target enzymes. WaterMap analysis calculated the thermodynamic properties of the hydration site in the binding pocket that significantly affects the biological activity. Loading **M3** on zinc oxide nanoparticles (ZnO NPs) increased the antiviral activity of the compound 1.5-fold, while maintaining a higher safety profile. In conclusion, lead optimized discovery following an iterated virtual screening in association with molecular docking and biological evaluation revealed a novel compound named **M3** with promising dual activity against SARS-CoV-2. The compound deserves further investigation for potential clinical-based studies.

## 1. Introduction

Severe acute respiratory syndrome coronavirus 2 (SARS-CoV-2) has caused an ongoing pandemic, as declared by the World Health Organization (WHO) on 11 March, 2020 [1]. It has become a global crisis with a severe impact on global public health and the economy, with unprecedentedly high mortality rate and ease of transmission [1]. Scientists have been working on developing vaccines and treatments to hinder the pandemic’s progress and lessen the disease’s risk factors. While several vaccines were recently granted emergency use authorization by the FDA, struggling to develop an effective drug against SARS-CoV-2 continues, as it is unlikely that vaccines alone can cease the pandemic because of continuous viral mutations [2,3,4]. In order to develop an anti-SARS-CoV-2 drug, it is necessary to identify possible drug targets for effective treatment with limited toxicity. The spike viral protein, the viral RNA-dependent RNA polymerase (RdRp), the viral main protease (M^pro^), the viral papain-like protease (PLpro) and the human furin protease and human transmembrane serine protease 2 (TMPRSS2) are potential targets for developing anti-SARS-CoV-2 drugs [5]. Until now, the only drug with a direct action on viral replication by inhibiting RdRp protein function has been remdesivir. It received an emergency use authorization from the FDA, although some studies have shown that remdesivir has no benefits compared to placebo in treating SARS-CoV-2 infection [6]. Some other drugs are still in clinical trials, such as AT-527, EIDD-2801, favipiravir, PF-07321332, PF-07304814, and niclosamide [4,7,8,9,10]. However, all the drugs mentioned are focused on either RdRp or M^pro^. With the recent emergence of several SARS-CoV-2 variants from other continents (the UK, South Africa, Brazil, and India variants) [11], it is important to develop a potential candidate with multiple targeting activities.

Recently, we discovered that **Compound 13** is a promising anti-SARS-CoV-2 treatment through dual targeting the activity of SARS-CoV-2 M^pro^ and human furin proteases [12]. The discovery of the compound was made following a virtual screening of 500,000 compounds, available in databases in association with molecular docking studies, enzyme inhibition screening assay, and in vitro confirmation [12]. Herein, **Compound 13** was used as a lead to discover a compound with more efficient binding activity against SARS-CoV-2 M^pro^ following iterated virtual screening. This was followed by filtering the selected compounds against TMPRSS2 for potential dual activity. The compounds showing potential shared binding activity were purchased and evaluated by in vitro antiviral activity and enzyme inhibition assay.

The limited bioavailability of some potential anti-SARS-CoV-2 drugs, such as PF-00835231 and GC-376, has diminished their efficacy in the clinical trials [10]. Therefore, zinc oxide nanoparticles (ZnO NPs) were employed in this study as a drug carrier [13] to enhance the bioavailability and efficacy of the selected compound.

The high mutation rate of SARS-CoV-2 encourages the use of a combination therapy or dual inhibitor drugs to combat the emerged viral resistance; however, the advantages of a dual inhibitor drug over a combination therapy can include (i) reducing the number of administered drugs, dosing, toxicity, and drug–drug interactions [14,15]; (ii) increasing patient compliance and avoid patient confusion; (iii) enhancing the sustained release of the drug; and (iv) reducing the probability of drug resistance and providing a platform for easier modification for possible future outbreaks [16]. A dual inhibitor approach has been developed against HIV [17] and influenza viruses [18] and showed great activity when compared to FDA-approved antiviral drugs. Furthermore, the encapsulation of a dual inhibitor drug over the formulation of two independent drugs adds more advantages as combination regimens often suffer from variations in the formulation properties, such as (i) particle size and surface charge; (ii) physicochemical properties may effect on formulation, handling, testing the activities in vitro and in vivo; (iii) solubility issue during formulation and bioavailability testing; and (iv) variable pharmacokinetics among different drugs [19].

This work presents the discovery of novel compounds with dual activity against critical SARS-CoV-2 targets, including viral and human proteases, with a proven efficacy in vitro and limited toxicity on normal human cells.

## 2. Results

### 2.1. Rational Lead Discovery of Potential Anti-SARS-CoV-2

**Figure 1:**
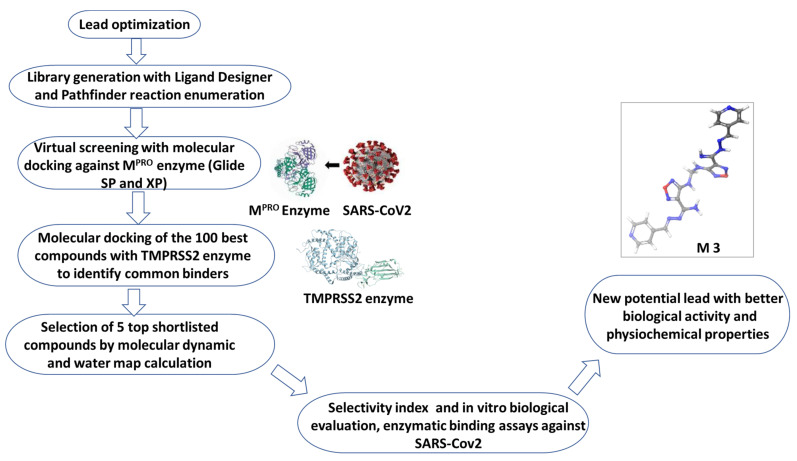
Flowchart of rational lead design and drug discovery of **M3**.

**Compound 13** showed promising anti-SARS-CoV-2 activity by dual-targeting the activity of M^pro^ and human furin protease [12], and was employed here as a lead for the discovery of a novel compound with efficient enzyme binding inhibition activity and limited toxicity (Figure 1). The lead optimized discovery depended on the presence of substitutions that can improve the efficacy, enhance the physicochemical properties, limit the off targeting, increase the solubility, and improve the overall pharmacokinetic activity. This was achieved by combining the superior performance of enumerated Ligand Designer tool and the PathFinder reaction enumeration workflow. The combined tools assisted in the iterated annotation of the compound library that can be easily synthetized and conform the 3D geometric structures of target enzymes (Figure 1).

**Figure 2:**
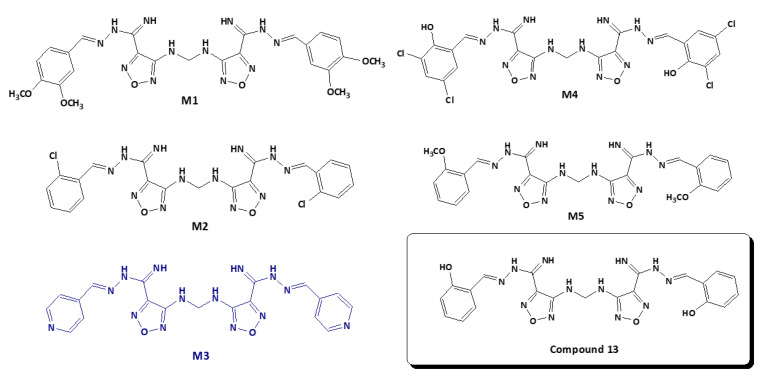
Chemical structures of the shortlisted compounds **M1–5** and **Compound 13** with potential inhibitory activity to SARS-CoV-2.

Briefly, a library of 2000 compounds was filtered following **Compound 13**-based iterated virtual screening against SARS-CoV-2 M^pro^ enzyme (PDB: 6LU7) using molecular docking studies. The 100 best molecules that satisfied the constraints and the fitting of the desired enzyme binding pocket space were retained. The 100 selected compounds with unique structural features were re-screened against the human TMPRSS2 (PDB ID: 2OQ5) enzyme using molecular docking studies. The best 20 shared compounds against both targets were further elaborated using visual inspection of its position and binding interactions (Supplementary Table S1 and S2). This optimized lead discovery process resulted in the selection of five compounds (**M1–5**) with potential binding activity to both viral targets (Figure 2 and Table 1). Consequently, compounds **M1–5** were investigated using integrated WaterMap analysis and MD tools to ensure their structure superiority, stability, and binding stability. Compounds **M1–5** were purchased and evaluated in vitro against SARS-CoV-2 and by enzyme inhibition assay in comparison to the parent, **Compound 13**.

### 2.2. Candidate Election by Enumerated Structure Ligand Designer

**Figure 3:**
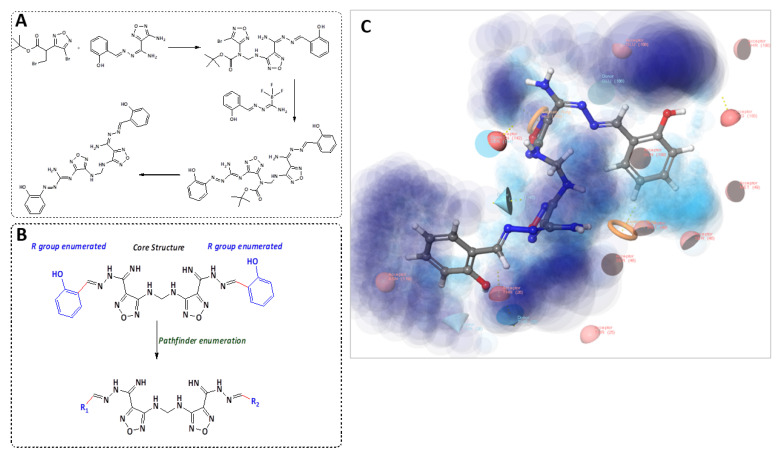
Enumerated structure design by PathFinder. (**A**) Proposed synthetic route for the synthesis of **Compound 13**. (**B**) R-group enumeration. (**C**) Ligand designer of **Compound 13** with M^pro^ binding pocket. Light blue shows the cavity space and dark blue shows the solvent exposure workflow.

To identify the novel ligands derivatives from **Compound 13**, PathFinder was used to predict the route of synthesis of **Compound 13** and to enumerate the commercially available building blocks of the compound (Figure 3A). The predicted synthetic steps were alkylation of amine followed by Suzuki coupling of the Boc-protected amine and subsequent deprotection. For instance, the novel ligands derivatives were generated by enumerating the building blocks using PathFinder (Figure 3B). The Ligand Designer tool was also used for 3D visualization of the enumerated structures, to analyze the workspace substation, and to select the pharmacophoric features of the predicted inhibitors within SARS-CoV-2 M^pro^ (Figure 3C). Diverse structures were enumerated with various R-substituted phenyl ring at different positions and an isosteric replacement of the phenyl ring with a heterocyclic ring. Examples of the R groups included various halogen substitutions, substituted amino groups, alkoxy, and alkyl groups. The enumerated compounds were further filtered out using PAINS offenders. The top 100 compounds that showed the best binding interaction with the M^pro^ enzyme (PDB: 6LU7) were selected. The compounds were further re-screened against TMPRSS2 (PDB ID: 2OQ5) enzyme for the selection of the 20 shared compounds with the highest binding to both protein targets (Supplementary Tables S1 and S2). This was followed by careful visual inspection of the binding interaction, which led to the selection of five compounds, **M1–5**. Further confirmation of the stability of **M1–5**-docked complexes with MD simulation was performed to overcome the loophole of the dynamic nature of the protein.

### 2.3. Two Candidate Compounds (***M3*** and ***M5***) Showed Promising Anti-SARS-CoV-2 Activity In Vitro

**Figure 4:**
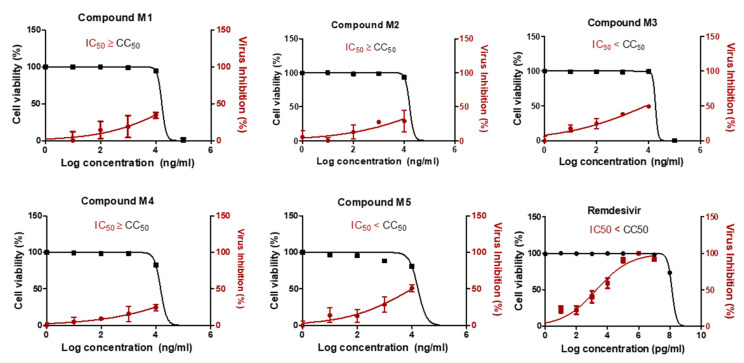
In vitro antiviral activity of compounds **M1–M5**. The antiviral activities of the compounds were screened on NRC-03-nhCoV strain (Accession # EPI_ISL_430820) co-cultured with Vero-E6 cells. To determine the most effective and safer compounds, selectivity index (SI) was measured, and the compounds with high SI were selected. Values of inhibitory concentration 50% (IC_50_) on viral cells and cytotoxic concentration 50% (CC_50_) on Vero cells were calculated using nonlinear regression analysis by plotting log inhibitor concentration versus normalized response (variable slope). The data display the mean of cell viability percentage ± SEM of 4 replicas.

Compared to **Compound 13**, the in vitro anti-SARS-CoV-2 activity of compounds **M1–5** revealed that only **M3** and **M5** showed a high selectivity index for antiviral activity relative to Vero-E6 cells toxicity with 50% cytotoxic concentration (CC_50_) higher than the IC_50_ by 2.3-fold and 1.7-fold, respectively (Figure 4). The IC_50_ of compounds **M3** and **M5** were 0.016, and 0.019 µM, respectively. The **M3** compound showed improved IC_50_ against SARS-CoV-2 compared to the previously reported IC_50_ of **Compound 13**. However, its IC_50_ was lower than that of remdesivir as a positive control.

**Figure 5:**
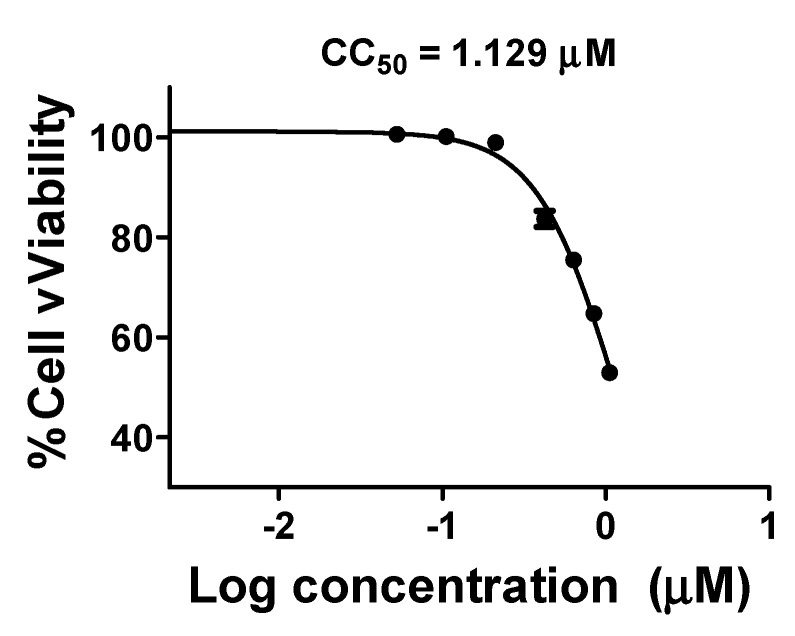
Cytotoxic activities of **M3.** The cytotoxic activity of **M3** compound was tested on normal human cells at different concentrations (0.105, 0.211, 0.422, 0.632, 0.843, and 1.054 µM). The data display the mean of cell viability percentage ± SEM of 3 replicas.

Because **M3** showed potent activity with a higher selectivity index, its cytotoxic activity was further tested on HDF cells using MTT assay. Compound **M3** (CC_50_ 1.129 µM ± 0.0019 was safe on human cells at ~70-time its IC_50(SARS-CoV-2)_ (Figure 5).

### 2.4. Compound ***M3*** Possesses Dual Inhibition Activity against SARS-CoV-2 by Targeting Both the Viral and Human Proteases

**Figure 6:**
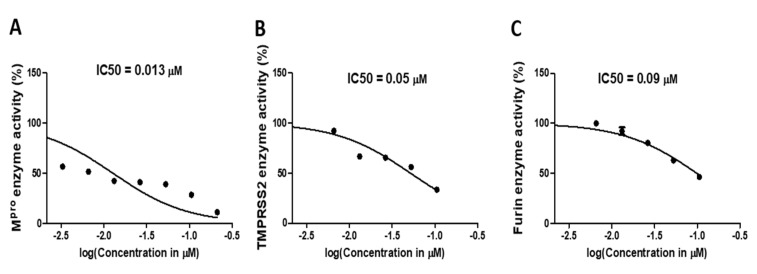
Inhibition activity of **M3** against selected critical SARS-CoV-2 targets. (**A**). IC_50_ calculation of **M3** against SARS-CoV-2 M^pro^ enzyme. (**B**) IC_50_ calculation of **M3** against human TMPRSS2 enzyme. (**C**) IC_50_ calculation of **M3** against human furin enzyme. The data display the mean of the percentage of the enzyme inhibition ± SEM of 3 replicas.

Enzyme inhibition assay showed that compound **M3** notably inhibited the activity of SARS-CoV-2 M^pro^ enzyme with an IC_50_ equal to 0.013 ± 0.003 µM (Figure 6A). The inhibition activity of compound **M3** was further tested against human TMPRSS2 protease. Interestingly, **M3** exhibited significant inhibition activity at IC_50_ 0.05 ± 0.002 µM (Figure 6B). Compound **M3** was further validated against furin protease, resulting in a potent activity at IC_50_ 0.09 ± 0.002 µM, 3.3-fold of the parent, **Compound 13** (Figure 6C).

### 2.5. Molecular Docking Indicated the Binding Efficiency of ***M3*** with SARS-CoV-2 M^pro^ and TMPRSS2 Protease

**Figure 7:**
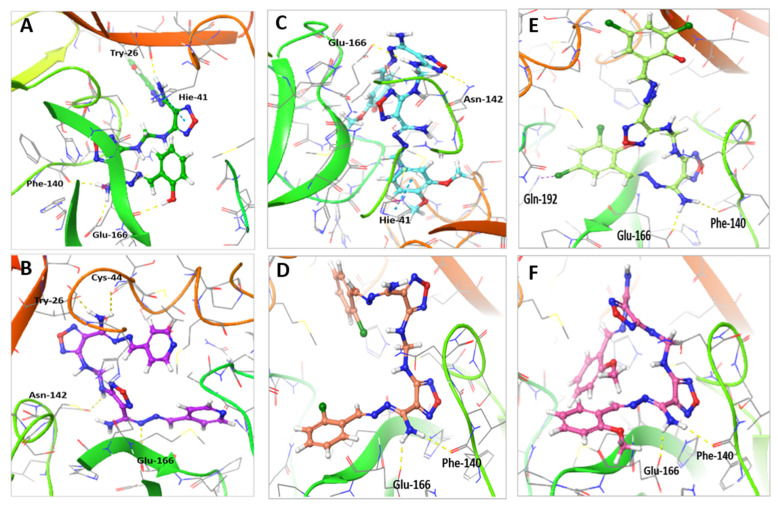
Molecular docking of compounds **M1–5** compared to **Compound 13** with SARS-CoV-2 M^pro^ (PDB: 6LU7). (**A**) The interaction of **Compound 13** (green stick) within the active site of M^PRO^. (**B**) The interaction of **M3** (purple stick) within the active site of M^pro^. (**C**) The interaction of **M1** (cyan stick) within the active site of M^PRO^. (**D**) The interaction of **M2** (orange stick) within the active site of M^pro^. (**E**) The interaction of **M4** (light green stick) within the active site of M^pro^. (**F**) The interaction of **M5** (pink stick) within the active site of M^pro^.

Molecular docking study of **M1–5** compared to **Compound 13,** indicated that the interaction of all compounds, except **M3**, showed a similar binding position to SARS-CoV-2 M^pro^ (Table 2 and Figure 7). The binding of **Compound 13** within the active site of M^pro^ enzyme showed four H-bonds with Glu-166, Phe-140 and Thr-26 residues along with a pi–pi stacking interaction with His-41 amino acid residue and a hydrophobic interaction with Met-49, Cys-44, and Met-165 residues (Figure 7A). On the other hand, the binding of **M3** within the active site of the M^pro^ enzyme showed a strong interaction made of four H-bonds with Cys-44, Glu-166, Thr-26, and Asn-142 amino acid residues and hydrophobic interactions with Met-44, Cys-44, Glu-166, and Met-165 residues (Figure 7B). Compound **M1** showed two H-bonds with Glu-166 and Asn-142 and pi–pi stacking interaction with His-41 and hydrophobic interaction with Met-49, Cys-145, Pro-168, and Met-165 residues (Figure 7C); **M2** showed two H-bonds with Glu-166 and Phe-140 and hydrophobic interaction with Met-49, Cys-145, Gln-189, and Met-165 residues (Figure 7D); **M4** showed two H-bonds with Glu-166 and Phe-140 and halogen bond with Gln-192 and hydrophobic interaction with Met-49, Cys-145, Gln-189, Gln-192, and Met-165 residues (Figure 7E); and **M5** showed two H-bonds with Glu-166 and Phe-140 residues and hydrophobic interaction with His-41, Met-49, Cys-44, Gln-189, and Pro-168 residues (Figure 7F).

**Figure 8:**
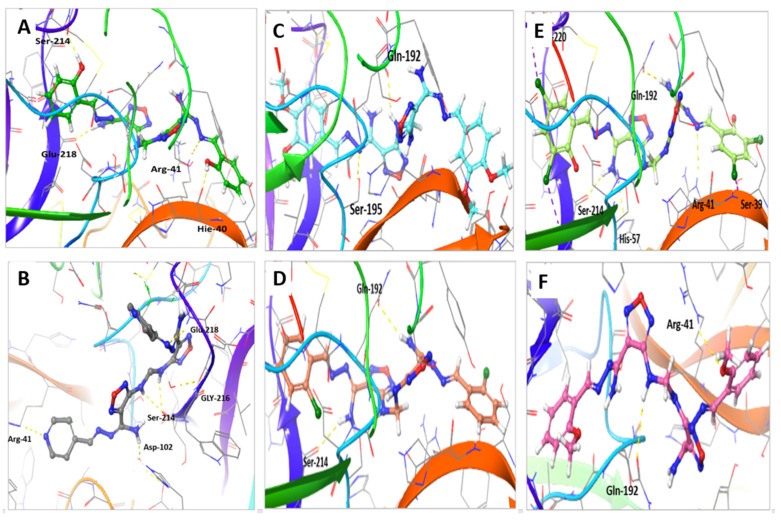
Molecular docking of compounds **M1–5** compared to **Compound 13** with TMPRSS2 (PDB:2OQ5) protein. (**A**) The interaction of **Compound 13** (green stick) within the active site of TMPRSS2. (**B**) The interaction of **M3** (gray stick) within the active site of TMPRSS2. (**C**) The interaction of **M1** (cyan stick) within the active site of TMPRSS2. (**D**) The interaction of **M2** (orange stick) within the active site of TMPRSS2. (**E**) The interaction of **M4** (light green stick) within the active site of TMPRSS2. (**F**) The interaction of **M5** (pink stick) within the active site of TMPRSS2.

The interaction of shortlisted compounds to TMPRSS2 in comparison to **Compound 13** was also calculated and showed the unique binding of **M3** (Table 3 and Figure 8). The binding of **Compound 13** with TMPRSS2 active sites showed four H-bonds with Arg-41, Ser-214, Glu-218, and His-40 amino acid residues, in addition to hydrophobic interaction with Gln-192, Arg-41, Ala-190, Cys-219, Try-36, and Val-213 amino acid residues (Figure 8A). On the other hand, the interaction of **M3** with TMPRSS2 showed six H-bonds with Arg-41, Ser-214, Glu-218, Gly-216, and His-99 amino acid residues, in addition to hydrophobic interactions with His-57, Arg-41, and Cys-219 amino acid residues (Figure 8B). **M1** showed two H-bonds with Ser-195 and Glu-192, and hydrophobic interaction with Arg-41, Cys-219, Try-149, and Val-213 amino acid residues (Figure 8C); **M2** showed two H-bonds with Ser-214 and Glu-192, and hydrophobic interaction with Arg-41, Cys-219, Try-36, Gln-192, and Val-213 amino acid residues (Figure 8D); **M4** showed four H-bonds with Ser-39, Glu-192, His-57, and Arg-41 and halogen bond with Ser-214 and Ala-220, and hydrophobic interaction with Arg-41, Cys-219, Try-228, and Val-213 amino acid residues (Figure 8E); **M5** showed two H-bonds with Arg-41 and Glu-192, and hydrophobic interaction with Arg-41, Cys-219, Try-149, Ala-190, and Val-213 amino acid residues (Figure 8F).

The results from molecular docking indicated that the docking pose of **M3** exhibited stable binding at the active sites of SARS-CoV-2 M^pro^ and human TMPRSS2 enzymes.

### 2.6. MD Simulation Confirmed the Stability of ***M3*** Complex with the Target Enzymes, SARS-CoV-2 M^pro^ and Human TMPRSS2

**Figure 9:**
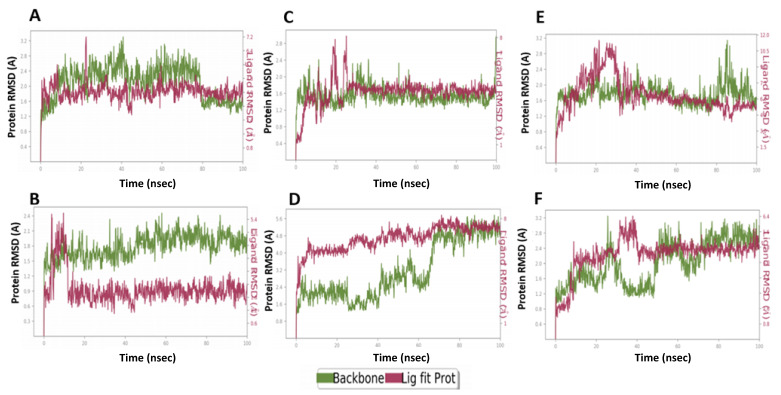
The root mean square deviation (RMSD) plots of compounds **M1–5** compared to **Compound 13** with SARS-CoV-2 M^PRO^ (PDB: 6LU7). (**A**) RMSDs of **Compound 13**. (**B**) RMSDs of **M3**. (**C**) RMSDs of **M1**. (**D**) RMSDs of **M2**. (**E**) RMSDs of **M4**. (**F**) RMSDs of **M5**. Green represents the protein backbone fluctuations, while the red represents the ligand fluctuations.

MD simulations for the shortlisted compounds within the active site of M^pro^ and TMPRSS2 enzymes indicated the superiority, stability, and stability of the **M3** complexes with both enzymes (Figure 9). MD results verified the stability of the compounds based on the fluctuation of root mean square deviation (RMSD) during the simulation. The ligand and protein RMSD were independently measured, where 2Å indicates the stability of the complex. The inhibitory activity was related to the decrease in residues fluctuation within the pocket site.

**M3** showed a higher binding stability with catalytic site residues of M^pro^ compared to **Compound 13**. The RMSD of **Compound 13** complex with M^pro^ was 1.9 Å compared to 1.6Å for the protein alone (Figure 9A). **Compound 13** showed a conformational change that lasted for 15 ns to reach the equilibrium (Figure 9A). Similarly, the **M3** complex reached the equilibrium after 15 ns, but showed a lower fluctuation range of 1.1Å (Figure 9B), indicating the stability of a formed complex over the simulations period (Figure 9B).

**M1** complex with M^pro^ showed a fluctuation that lasted for a longer time (25 ns) until reaching equilibrium with RMSD range of 1.2Å compared to 0.7 Å for the protein alone (Figure 9C). **M2** protein complex showed higher fluctuation range at 1.3 Å when compared to 1.6 Å for the protein alone and it stabilized after a longer time (60 ns) (Figure 9D). **M4** complex required 35 ns to reach the equilibrium and with a relative stability range of 2.1 Å when compared to 1.5 for the protein (Figure 9E). **M5** complex required a somewhat longer time for stabilization at 40 ns with a RMSD value of 1.6 Å compared to 1.5 Å for the protein (Figure 9F). This indicated the superiority of the **M3** complex during the MD simulation and the highest stability of its complex, **M2** showed the lowest stability, while **Compound 13** showed moderate stability and **M1** and **M5** required a longer time for stabilization.

**Figure 10:**
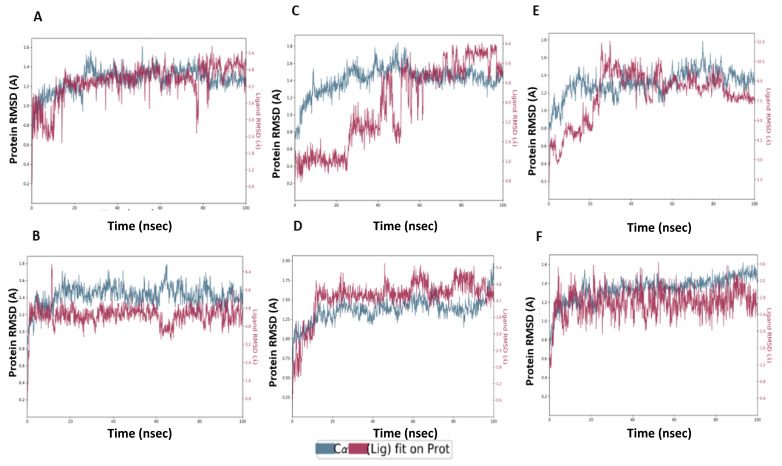
The root mean square deviations (RMSD) plots of compounds **M1–5** compared to **Compound 13** with human TMPRSS2 (PDB ID: 2OQ5). (**A**) RMSDs of **Compound 13**. (**B**) RMSDs of **M3**. (**C**) RMSDs of **M1**. (**D**) RMSDs of **M2**. (**E**) RMSDs of **M4**. (**F**) RMSDs of **M5**. Green represents the protein backbone fluctuations, while the red represents the ligand fluctuations.

Regarding the binding with TMPRSS2, **Compound 13** complex showed fluctuation for the first 25 ns until it reached equilibrium. The obtained RMSD range was 2.4 Å compared to 0.7 for the protein (Figure 10A). **M3** complex with TMPRSS2 reached equilibrium after 15 ns and showed low protein and ligand fluctuation of RMSD ranges of 0.5 and 1.8 Å, respectively, indicating the stability of **M3** complex over the MD simulation (Figure 10B). **M1** protein complex required a longer time (60 ns) to reach equilibrium and showed protein and ligand RMSD fluctuation ranges of 0.6 and 2.1 Å, respectively (Figure 10C). **M2** protein complex showed fluctuation of the protein for the first 20 ns to reach the equilibrium with the protein, and the ligand RMSD range was 0.7 and 1.9 Å, respectively (Figure 10D). **M4** complex required 30 ns to reach the equilibrium, after that, it showed a relative stability with RMSD values of the protein and ligand ranging from 0.8 and 2.1 Å, respectively (Figure 10E). **M5** complex showed certain stability over MD simulation. It required 20 ns for equilibrium with RMSD values of protein and ligand ranging from 0.5 and 1.7 Å, respectively (Figure 10F). Collectively, it is obvious that the **M3** protein complex showed stability over the MD simulation, better than the **Compound 13** complex (Figure 9 and Figure 10).

**Figure 11:**
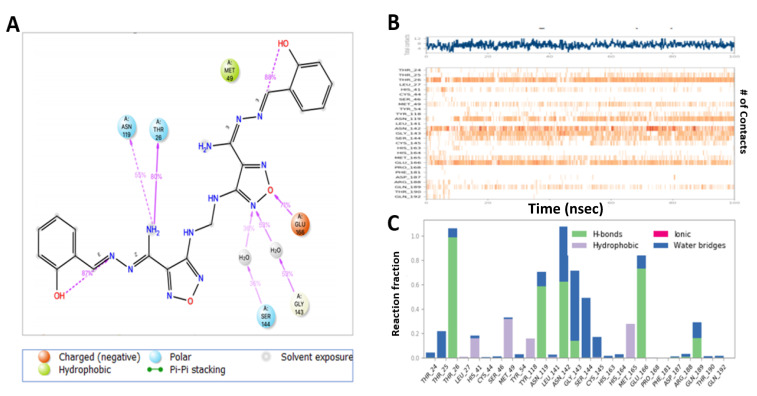
Interaction diagram of **Compound 13** with M^pro^ observed during the molecular dynamic simulation. (**A**) The protein–ligand interaction diagram. (**B**) The top panel showed the specific contact of M^pro^ protein with **Compound 13** in each trajectory course, the bottom panel showed the amino acid residues that interact with the ligand in the trajectory time frame. The residues making more than one contact were shown in a darker color shade. (**C**) Schematic diagram of ligand interaction with the amino acid residues of protein during MD simulation.

**Compound 13** complex with M^pro^ exhibited H-bond formation with NH_2_ moiety of ligand with Thr-26 (80%), Asn-119 (55%), N of the oxadiazole ring with Gly-143 (59%, 59%) and Ser-144 (30%, 36%) via a water bridge, O of the oxadiazole ring with Glu-166 (71%) (Figure 11A), and hydrophobic contacts with His-41, Met-49, Tyr-119, and Met-164, whereas Thr-61, Tyr-94, Asp-37, Cys-191 Phe-141, and Cys-191 constituted a more flexible region (Figure 11B,C).

**Figure 12:**
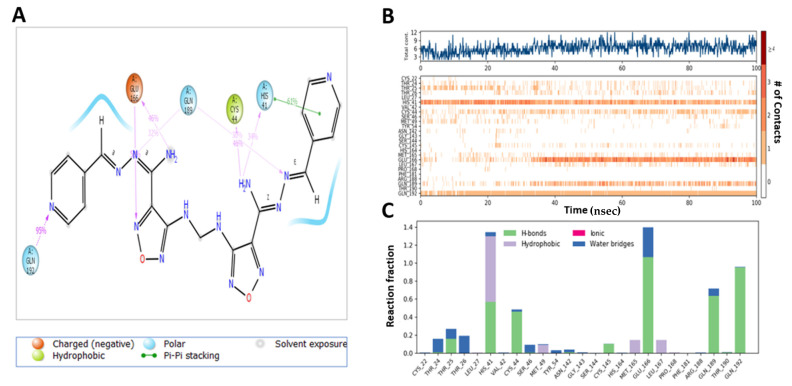
Interaction diagram of **M3** with M^pro^ observed during the molecular dynamic simulation. (**A**) The protein–ligand interaction diagram. (**B**) The top panel showed the specific contact of M^pro^ protein with **M3** in each trajectory course, the bottom panel showed the amino acid residues that interact with the ligand in the trajectory time frame. The residues making more than one contact were shown in a darker color shade. (**C**) Schematic diagram of ligand interaction with the amino acid residues of protein during MD simulation.

MD analysis of the **M3** complex with M^pro^ showed H-bond interactions similar to those predicted by the docking study (Figure 12A). **M3** exhibited H-bond interactions between the NH_2_ of the ligand with His-41 (34%) and Cys-44 (30%), and Glu-166 (46%), N with Gln-189 (46%), N of oxadiazole with Glu-166 (58%), and N of pyridine with Gln-192 (95%), and pi–pi stacking with His-41(61%) and hydrophobic interaction with His-41, Met-49, Met-165, and Leu-167. The region made of Leu-27, Ser-144, His-164, Phe-181, and Ala-191 residues was more flexible and had no hydrogen bond and hydrophobic interactions (Figure 12B,C).

**Figure 13:**
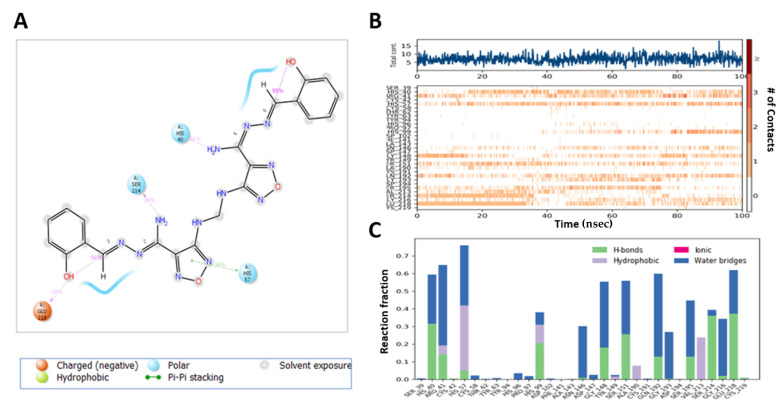
Interaction diagram of **Compound 13** with TMPRSS2 observed during the molecular dynamic simulation. (**A**) The protein–ligand interaction diagram. (**B**) The top panel showed the specific contact of TMPRSS2 protein with **Compound 13** in each trajectory course, the bottom panel showed the amino acid residues that interact with the ligand in the trajectory time frame. The residues making more than one contact were shown in a darker color shade. (**C**) Schematic diagram of ligand interaction with the amino acid residues of protein during MD simulation.

**Compound 13**-induced docked complex with TMPRSS2 showed similar modes of binding. **Compound 13** exhibited an H-bond between NH_2_ moiety of ligand with His-40 (54%), Ser-214 (36%), and OH with Glu-218 (34%) (Figure 13A). Hydrophobic interactions of the oxadiazole ring with His-57, His-99, Tyr-149, Ala-190, and Val-213 were stable, whereas Thr-61, Tyr-94, Asp-37, Cys-191 Phe-141, and Cys-191 regions were more flexible (Figure 13B,C).

**Figure 14:**
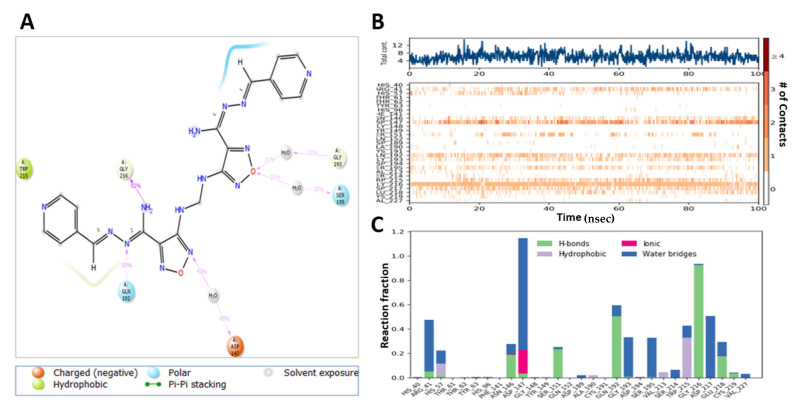
Interaction diagram of **M3** with TMPRSS2 observed during the molecular dynamic simulation. (**A**) The protein–ligand interaction diagram. (**B**) The top panel showed the specific contact of TMPRSS2 protein with **M3** in each trajectory course, the bottom panel showed the amino acid residues that interact with the ligand in the trajectory time frame. The residues making more than one contact were shown in a darker color shade. (**C**) Schematic diagram of ligand interaction with the amino acid residues of protein during MD simulation.

MD analysis of the induced docking fit pose of **M3** complexed with TMPRSS2 exhibited a water bridge H-bond of oxadiazole oxygen with Gly-193 (30%, 35%) and Ser-195 (33%, 30%); ionic interaction of oxadiazole N with Asp-147 (43%, 43%); hydrogen bond interaction of NH_2_ with Gly-216 (92%); and N interaction with Gln-192 (50%) (Figure 14A). Hydrophobic interactions with His-57, Ala-190, Val-213, and Typ-215 were in the stable region, whereas the region made of Thr-61, Phe-141, Gln-152, and Cys-191 was more flexible and had no hydrogen bond and hydrophobic interactions (Figure 14B,C). This indicates the higher stability of **M3**–protein complexes with M^pro^ and TMPRSS2 catalytic site residues when compared to **Compound 13**.

MD simulation of **M1** with M^pro^ binding site showed H-bond interaction of NH_2_ moiety with Glu-166, NH with Asn-142, N of the oxadiazole with His-163 and with Glu-166 through a water bridge (Supplementary Figure S3A). Further, it showed hydrophobic contacts with Thr-26, Leu-27, His-41, Met-49, and Cys-145, whereas the region made of Thr-26, Leu-50, Asn-119, Phe-140, and Arg-188 was more flexible (Supplementary Figure S3B,C). MDS of **M2** complex with M^pro^ showed H-bonding of oxadiazole N with Glu-166, Gly-143, N with Gln-189 and a halogen bond with His-4 (Supplementary Figure S4A), hydrophobic contacts with Leu-27, His-41, Cys-44, Met-49, and Met-165, whereas His-172 and Pro-168 constituted a more flexible region (Supplementary Figure S4B,C). MD simulation of **M4** complex with M^pro^ exhibited ionic interactions of oxadiazole N and O with His-41 and Cys-44 and N of oxadiazole with Ser-46 (Supplementary Figure S5A); in addition to ionic contacts with Thr-25, Met-49, and Gln-189, the hydrophobic contacts with His-41, Met-49, Met-165, and Pro-168. Gln-192 and Ala-191 constituted a more flexible region with no observed interactions (Supplementary Figure S5B,C). **M5-**M^pro^ complex showed hydrogen bonding of NH_2_ moiety with Glu-166, Asn-142 through a water bridge and N of the oxadiazole with Gly-143 (Supplementary Figure S6A); in addition to hydrophobic interaction with Thr-26, His-41, Cys-44, Met-49 and Met-165. His-172, His-164, Gln-192, and Pro-52 constituted a more flexible region (Supplementary Figure S6B,C).

**M1** complex with TMPRSS2 showed H-bonding of NH_2_ moiety with Gly-216, Gly-148, N of oxadiazole with Arg-41 through a water bridge and hydrophobic interactions of the oxadiazole ring with His-57 (Supplementary Figure S7A). Further, hydrophobic contacts with His-40, Gly-148, Asp-189, Trp-215, and Val-213 constituted the stable region, whereas Thr-61, Lys-145, Cys-191, Asp-37, and Cys-219 was the flexible region (Supplementary Figure S7B,C). Compound **M2** complex with TMPRSS2 showed hydrogen bonding of NH_2_ moiety with Ser-214 and His-57 (Supplementary Figure S8A) and hydrophobic interactions of the oxadiazole ring with His-57. Hydrophobic contacts with Arg-41, His-99, Ala-190, Tyr-149, and Val-213 constituted the stable region, whereas regions Asn-146, Asp-217 and Glu-218 were more flexible (Supplementary Figure S8B,C). MD analysis of the inhibitor **M4** with TMPRSS complex exhibited an H-bond of methoxy oxygen with Gln-192 and ionic interaction with Arg-41, NH_2_ with Gly-148, Gln-192 and ionic interaction with Asp-147 (Supplementary Figure S9A). The region made of Gly-38, Cys-58, Lys-76, His-96, Phe-141, Asn-146, Gly-216, and Asp-217 was more flexible and with no interactions (Supplementary Figure S9B,C). **M5**–TMPRSS2 complex showed hydrogen bonding of NH_2_ moiety with Gly-148, Ser-214, and His-57, N of the oxadiazole with Asp-147 through water bridge, N and methoxy showed interaction with Arg-41 through water bridge, N with Gly-216 through a water bridge (Supplementary Figure S10A). Ala-190 and Cys-42 constituted a more flexible region (Supplementary Figure S10B,C).

### 2.7. WaterMap Analysis Showed the Pronounced Efficacy of ***M3***

**Figure 15:**
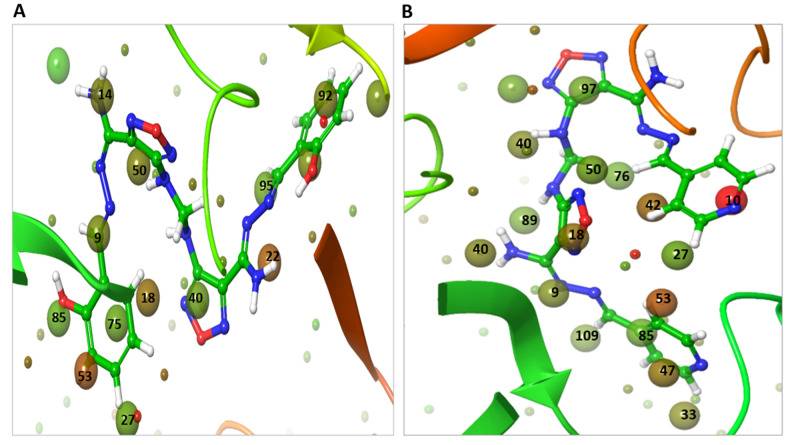
WaterMap analysis of the hydration sites of M^pro^ binding pocket with the docked of pose compounds. (**A**) The overlay and displacement of unstable and replaceable water with **Compound 13**. (**B**) The overlay and displacement of unstable and replaceable water with **M3.** The hydration sites were shown with small spheres. The color spectrum ranged from red (most unfavorable) to green (least unfavorable).

WaterMap analysis of M^pro^ binding site showed 111 hydration sites with a sum up free energy of 233.02 kcal/mol, indicating that the site has strong potential for ligand binding pockets (Supplementary Table S3). The number of replaceable water molecules at the dG cut off value −2.0 to +2.0 kcal/mol was 60 kcal/mol. The number of unstable water molecules with dG cut off value > 2.0 kcal/mol was 51 kcal/mol. There was no stable water with dG value < −2.0 kcal/mol (Supplementary Table S3). The WaterMap analysis of the TMPRSS2 binding site showed 105 hydration sites with a sum up free energy 219.34 kcal/mol, indicating that the site has strong potential for ligand binding pockets (Supplementary Table S3). The number of replaceable water molecules was 59, while the number of displaceable unstable water molecules was 47 and there was no stable water. The docked pose of **Compound 13** showed displacement and replacement of the M^pro^ hydration site waters at 9, 14, 18, 22, 27, 35, 40, 50, 53, 55, 66, 75, 85, 89, 92, and 95 with a dG sum up of 29.95 kcal/mol (Figure 15A). On the other hand, **M3** showed displacement and replacement of 9, 10, 18, 27, 33, 40, 42, 47, 48, 50, 53, 76, 85, 89, 95, 97, and 109 with a higher dG sum up of 46.29 kcal/mol (Figure 15B). Two of the most unstable water (in red color) at sites 10 and 53 with dGs of 7.52 and 4.4 kcal/mol, respectively, were displaced by the pyridine moiety of **M3**. Furthermore, the least unstable water at sites 47 and 42 with dG of 3.15, and 3.65 kcal/mol, respectively, were displaced by the pyridine moiety of **M3**. The water at site 18 with dG 3.19 kcal/mol was displaced by the oxadiazole moiety. In the case of **Compound 13**, it did not displace any unstable water (in red color) at the binding site, instead, it displaced only less unstable water at 18, 22, and 53 sites with 3.19, 4.02, and 4.4 kcal/mol. This explains the improved potency of **M3** over the lead **Compound 13**.

**Figure 16:**
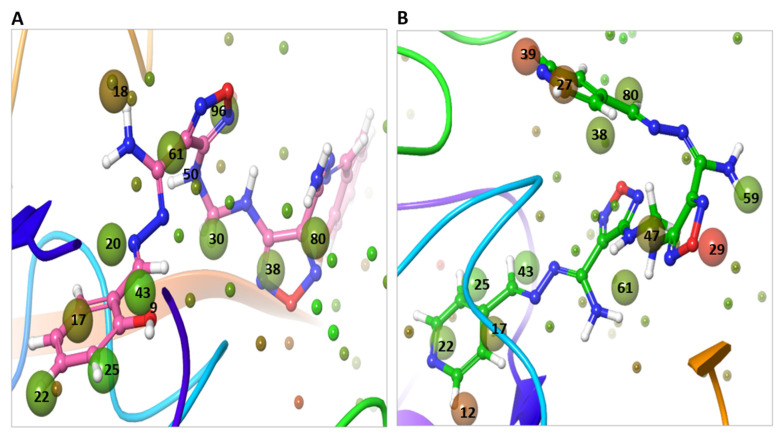
WaterMap analysis of the hydration sites of TMPRSS2 binding pocket with the docked pose of compounds. (**A**) The overlay and displacement of unstable and replaceable water with **Compound 13**. (**B**) The overlay and displacement of unstable and replaceable water with **M3**. The hydration sites were shown with small spheres. The color spectrum ranged from red (most unfavorable) to green (least unfavorable).

The WaterMap analysis of the docked position of **compound 13** within the TMPRSS2 binding site showed displacement and replacement of waters at hydration sites 17, 18, 22, 25, 30, 38, 43, 61, and 96, with dG 12.40 kcal/mol (Figure 16A). On the other hand, **M3** showed displacement and replacement of water at sites 14, 17, 22, 25, 27, 29 31, 38, 39, 43, 47, 59, 61, 77, and 80 with a higher dG, 34.53 kcal/mol (Figure 16B). The most unstable water (in red color) at sites 14, 27, and 39 with dG 4.45, 4.21, and 5.30 kcal/mol were displaced by the pyridine moiety of **M3**, while the water at site 29 with dG 6.58 kcal/mol was displaced by the oxadiazole moiety of **M3**. The less unstable water at site 18 with dG of 3.25 kcal/mol was displaced by **Compound 13**.

### 2.8. Adsorption of ***M3*** Compound on ZnO NPs Enhanced the Efficiency against SARS-CoV-2

**Figure 17:**
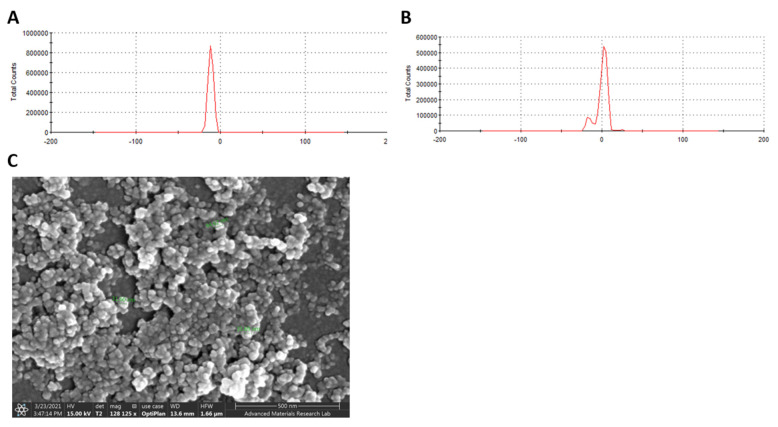
Characterization of compound-loaded ZnO NPs. Zeta potential measurement for (**A**) drug-free ZnO NPs and (**B**) ZnO NPs loaded with compound **M3**. NPs samples were diluted with distilled water and zeta potential were determined using Laser Doppler Velocimetry (LDV) mode. (**C**) SEM micrographs of ZnO NPs loaded with **M3**. ZnO NPs samples were placed on carbon tape after dryness then coated with gold prior to measurement.

**Figure 18:**
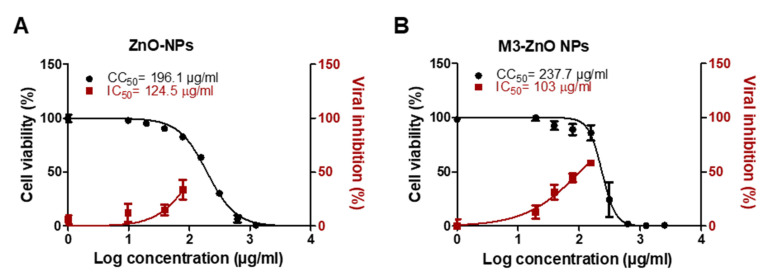
In vitro antiviral activity of (**A**) drug-free ZnO NPs compared to (**B**) **M3**-loaded ZnO NPs. The antiviral activity was assessed using NRC-03-nhCoV strain (Accession # EPI_ISL_430820) co-cultured with Vero-E6 cells. IC_50_ and CC_50_ on viral and Vero cells were calculated using nonlinear regression analysis by plotting log inhibitor concentration versus normalized response (variable slope). The data display the mean of cell viability percentage ± SEM of 4 replicas.

Compound **M3** was successfully adsorbed on ZnO NPs with an entrapment efficiency of 95%. The particle surface charge (Zeta potential) of ZnO NPs before and after **M3** loading was −11.3 ± 3.09 and 0.174 ± 7.38 mV, respectively (Figure 17A,B). The surface morphology of the **M3**-loaded ZnO NPs was well-defined spherical shaped NPs with smooth surfaces and without any surface cracks (Figure 17C). The particle size was < 50 nm (Figure 17C). Compared to drug-free ZnO NPs (Figure 18A), **M3**-loaded ZnO NPs showed significant antiviral activity at IC_50_ 103 µg/mL (Figure 18B). **M3**-loaded ZnO NPs showed a high selectivity index for antiviral activity relative to Vero-E6 cells toxicity with CC_50_ values higher than the IC_50_ values by 2.3-fold compared to 1.6 for the drug-free ZnO NPs. The developed ZnO NPs contained a lower concentration of **M3** (IC_50_ equal to 0.011 µM, which is ~30% lower than the IC_50_ of the compound alone) and showed a wider safety profile (CC_50_ equal to 237.7 µg/mL).

## 3. Discussion

While the number of confirmed infected cases and deaths due to COVID-19 exceeds ~208 and 4.4 million, respectively, as reported by the WHO on 15 August 2021, with an unprecedented increase worldwide [20], no drug is currently approved by the FDA for SARS-CoV-2 infection except for remdesivir, but with controversial activity [21]. Consequently, the discovery of drugs with antiviral adaptability is crucial. Several candidate drugs against SARS-CoV-2 were proposed, including 519 in the preclinical research and 419 in clinical trials according to April 2021 update [22]. By August 2021, the update of COVID-19 drug development showed (i) 81 antibodies; (ii) 31 antivirals; (iii) 6 RNA-based compounds; (iv) 34 cell-based compounds; and (v) 18 re-purposed compounds [23]. Besides supportive medications including bronchodilator and anti-inflammatory are under clinical trial for the final approval. The first re-purposed FDA drug was hydroxyl-chloroquine that is later discontinued by the WHO in June 2020 [24]. On March 2020, the center for disease control and prevention (CDC) considered remdesivir as an adaptive protocol for hospitalized COVID-19 patients [25]. Several antiviral drugs were investigated to treat COVID-19 disease and currently in phase III clinical trials such as favipiravir (April 2020) [26] and ritonavir/lopinavir (mid-2020) [27]. Novel antibodies, such as Casirivimab/imdevimab cocktail developed in (March 2020) [28] under the brand name REGEN-CoV, was intended to inhibit the mutational escape of the virus, a Bamlanivimab and etesevimab cocktail was granted an FDA emergency use authorization (EUA) approval in November 2020 [29] and Sotrovimab received FDA-EUA in May 2020 to treat moderate to severely infected COVID-19 patients [30]. In early 2021, Pfizer commenced phase I clinical trial for a novel protease inhibitor named PF-07321332 [31].

We have developed a novel anti-SARS-CoV-2 compound (**M3**) following an iterated virtual screening of our previously reported molecule (**Compound 13**) [12].

**Figure 19:**
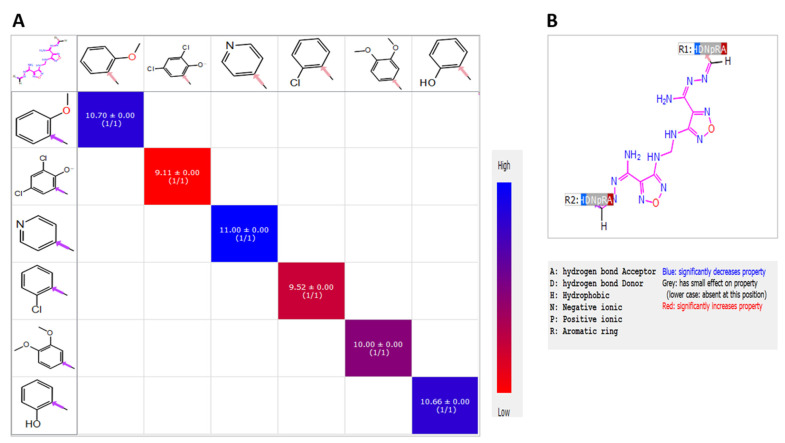
SAR Heatmap analysis. (**A**) PIC_50_ values were displayed as colors ranging from red to blue, as shown in the key. (**B**) QSAR analysis, and pharmacophoric features were displayed as colors of red, grey, and blue as shown in the key.

Structure–activity relationships (SAR) of shortlisted compounds identified following iterated virtual screening and molecular docking of **Compound 13** were studied using the R-group analysis tool. It showed that a small chemical structural change resulted in a substantial variance in the binding affinity and potency. The different R-groups orientations showed dissimilar experimental values of antiviral PIC_50_. The superior activity was indicated by the replacement of the 2-hydroxy phenyl moiety of **Compound 13** with the 4-pyridinyl moiety of **M3**. The replacement with 2-OCH_3_ phenyl of **M5** retained an almost similar activity of the lead **Compound 13**. On the contrary, the replacement with 2-chloro-phenyl of **M2** and the 3,5-dichloro-4-hydroxyphenyl moiety of **M4** exhibited a negative influence on the activity. The results from SAR heatmap analysis showed the variance activity with colors ranging from red to blue (Figure 19A). Moreover, the pharmacophoric R-QSAR analysis highlighted that the hydrogen bond acceptor (HBA) was significantly increased the activity (Figure 19B); **t**herefore, the chloro-substitution, the weak HBA group, of **M2** and **M5** showed decreased activity. Hence, the increased activity of **M3** and **M5** was due to the nitrogen- and oxygen-containing groups, better HBA groups, which would benefit future optimization. Furthermore, the difference in the structural activity between **M3** and **Compound 13** can be further explained by the incorporation of pyridine moiety in most of the hydrophobic and hydrogen bond interactions with the proteins binding sites that stabilized the complexes. For instance, WaterMap analysis showed that **M3** pyridine moiety displaced the most unfavorable hydration site of M^pro^ and TMPRSS2 proteins with total free energy of 18 and 14 kcal/mole, respectively, which eventually resulted in the significantly improved potency of **M3**.

The literature is rich with in silico studies for the discovery of molecules with potential activity against SARS-CoV-2 infection [32,33,34,35,36]; however, few studies employing enzyme inhibition assays and/or in vitro experiment supported in silico data. In the present study, compound **M3** showed promising in vitro activity against SARS-CoV-2 infected Vero-E6 cells with a potency that was seven-fold higher than the parent compound (**Compound 13**) and with higher CC_50,_ indicating the validity of the applied virtual screening methodology on developing new analogs with improved efficacy. Additionally, the in vitro antiviral activity of compound **M3** showed that it is more potent than many reported anti-SARS-CoV-2 compounds, despite the fact that some of the reported compounds are currently in clinical trials. For instance, the in vitro IC_50_ of remdesivir (FDA authorized), favipiravir (under clinical trial), and nafamostat against SARS-CoV-2 infected Vero-E6 cells are 0.77, 61.88, and 22.5 µM, respectively, as reported by Wang et al. [37]. Hung et al. assessed the in vitro anti-SARS-CoV-2 activity of the broad spectrum M^pro^ inhibitor (GC376) and the reported IC_50_ was 0.91 µM [21]. Furthermore, the IC_50__(SARS-COV-2)_ of niclosamide (currently under clinical trials) is 0.28 µM, as claimed by Jeon et al. [38].

The data obtained from the enzyme inhibition assay demonstrated the inhibition efficiency of **M3** against SARS-CoV-2 M^pro^, human TMPRSS2, and furin enzymes. The aforementioned enzymes are required by SARS-CoV-2 for viral replication and viral entry [39]. Generating compounds to shut down both the viral entry and replication and concurrently acting on viral and human therapeutic targets will boost and expand the arsenal against SARS-CoV-2 by adapting to the viral genetic variants that are currently emerging [39,40]. To initiate infection, SARS-CoV-2 has to attach viral spike proteins to cell receptors, which must be activated by host proteins (TEMPRSS2 and/or furin) in order to fuse with the host cell membrane [41]. Blocking both host proteins (TEMPRSS2 and furin) will prevent the SARS-CoV-2 infection even with viral genetic variants. On the other hand, SARS-CoV-2 replication requires the functional proteins nsp4 that is released through the cleavage of polyproteins pp1a and pp1ab [42]. This cleavage is mediated by M^pro^, and consequently, inhibiting M^pro^ hinders the ability of the virus to replicate within host cells [43]. Targeting M^pro^ can provide high selectivity as it has a preference for a glutamine substrate, which is missing in the host proteases [44].

The identified IC_50_ of **M3** against M^pro^ in the present study is 10-times lower than the parent compound (**Compound 13**) and even lower than the reported M^pro^ inhibitors in the literature. The most potent M^pro^ in the literature was reported by Ma et al. and employed the Selleckchem bioactive compound library to screen several protease inhibitors, and GC-376 was found to have a lower M^pro^ IC_50_ (0.03 µM) [44]. Li et al. measured the IC_50_ of 15 compounds selected based on prior virtual screening against M^pro^, which showed that the IC_50_ for the most potent compound (DIP) was 0.6 µM [45]. Another potent M^pro^ inhibitor (Calpain inhibitor X11) was presented by Sacco et al., with an M^pro^ IC_50_ equal to 0.453 µM [5]. Ebselen is a M^pro^ inhibitor that is acts at IC_50_ 0.67 µM [46]. Finally, Zhang et al. and Dai et al. designed M^pro^ inhibitors with IC_50_ values of 0.18, 0.053, and 0.04 µM for compounds 11r, 11a, and 11b, respectively [47,48].

Herein, we also employed ZnO NPs as a nano-therapeutic strategy to enhance the efficacy of compound **M3** against SARS-CoV-2 infection. ZnO NPs were selected due to their antiviral activity, ability to enhance drug solubility and bioavailability, cost-effective, biocompatibility with human cells and being approved by FDA as a pharmaceutical excipient [49,50,51,52]. The well-characterized and commercially available ZnO NPs were used in this study with claimed particle sizes of 10–30 nm and a spherical shape. Incubating compound **M3** with ZnO NPs under stirring resulted in a significant entrapment efficiency, which could be attributed to the ability of ZnO NPs to set strong electrostatic interactions, chelation, or covalent bonds with a wide range of molecules [53,54,55]. SEM results indicated that the size and shape of the obtained ZnO NPs were similar to the ones claimed by the vendor, while the zeta potential was lowered after incubating the **M3** compound with the ZnO NPs, confirming the successful loading of compound **M3** on the surface of the ZnO NPs. Our in vitro data indicated that the drug-free ZnO NPs at non-toxic concentrations possess antiviral activity against SARS-Cov-2 and loading the **M3** compound on ZnO NPs further enhanced the efficacy against SARS-CoV-2 infected cells. The antiviral activity of the ZnO NPs was reported against influenza virus (H1N1) and herpes simplex virus type 1 [52,56]. The direct antiviral activity of ZnO NPs can be attributed to the nano-size of the ZnO NPs, which enables the NPs to be passively internalized by the cell, together with the ability of ZnO NPs to release Zn^2+^ ions and reactive oxygen species (ROS) that can damage the lipids, proteins, and nucleic acids of the virus [57,58].

## 4. Conclusions

Several in silico studies have performed to identify compounds with potential antiviral activity against SARS-CoV-2; however, very limited numbers were biologically validated. This study employed novel computational techniques to carefully shortlist the compounds with potential binding affinity to important SARS-CoV-2 protein targets. Our previously identified **Compound 13**, which showed significant activity against SARS-CoV-2, was a subject of iterated virtual screening against SARS-CoV-2 M^pro^, in addition to R-group enumeration using Ligand Designer and PathFinder tools. The generated derivatives were re-screened against TMPRSS2, in addition to visual inspection leading to the identification of five shared compounds, with compound **M3** showing the most binding fit to both enzymes, confirmed by WaterMap analysis and molecular dynamic simulations. The results were validated by in vitro and enzyme inhibition assays, revealing that **M3** is a potent anti-SARS-CoV-2 when compared to the parent **Compound 13** and other antiviral drugs currently in the clinical trials. Future research studies are planned to overcome the limitations of computational modeling techniques, which may involve in vivo study and validation via crystallography and kinetic studies. The techniques employed in this study are the first, and consequently lead to the discovery of a potent compound, which deserves a careful look, either for further future optimization or clinical studies.

## 5. Experimental Section

### 5.1. Material

The compounds used in this study were purchased from https://mcule.com/ (accessed on 3 May 2021), including MCULE-3769246260, MCULE-2110509965, MCULE-8998847154, MCULE-1886473350, and MCULE-7013373725. Assay kits including 3CL Protease (3CL^pro^), Assay Kit (Cat# 78042-1), furin protease assay kit (Cat # 78040), TMPRSS2 fluorogenic assay kit (Cat# 78083) were purchased from BPS Bioscience, San Diego, CA, USA. ZnO NPs (99+%, 10–30 nm) was purchased from US Research Nanomaterials, Inc, Houston, TX, USA.

### 5.2. Computational Studies

All computational work was carried on Ubuntu desktop workstation in Intel^®^ Xenon^®^ Gold 6130 CPU @ 2.10 GHz × 64 processors, Quadro P620/PCle/SSE2 graphics card and 134.8 GB RAM on the Maestro graphical user interface of Schrödinger Suite 12.7 available at www.schrodinger.com (accessed on 3 May 2021).

#### 5.2.1. Protein Preparation

The 3D crystal structures of SARS-CoV-2 M^pro^ (PDB ID: 6LU7) and TMPRSS2 (PDB ID: 2OQ5) enzymes were downloaded from a protein data bank (https://www.rcsb.org/ (accessed on 3 May 2021)). The proteins were prepared and refined using the Protein Preparation Wizard Maestro to ensure structural correctness [59]. Crystallographic water molecules beyond 5Å were removed. All the missing hydrogen atoms were added at pH 7.3 for appropriate ionization and the tautomerization state of amino acid residues and proper bond order were assigned. Next, the refining of the protein structures was performed and the water molecules with <3 hydrogen bonds to non-waters were deleted. Finally, the energy minimization was done using OPLS4 to relieve the steric clashes [60].

#### 5.2.2. Ligand Preparation

The 2D structures of the generated library were converted to 3D structures using LigPrep (Schrodinger) [61]. Hydrogen atoms were added, and the salt ions were removed. The most probable ionization states were calculated at pH 7.3 using the Epik module [62,63]. During the ligand preparation, the specified chirality of the 3D crystal structure was retained. The subsequent energy minimization of each structure was carried out using the OPLS4 force field [60] and was filtered through a relative energy tool to exclude the high energy structures from the given input. Any errors in the ligands were eradicated in order to enhance the accuracy of the molecular docking [64].

#### 5.2.3. Grid Generation

The ligands in the crystal structure of M^pro^ and TMPRSS2 enzymes were used for grid generation. A grid box was generated at the centroid of the active site for docking studies, and the active site was defined around the ligand crystal structure.

#### 5.2.4. Molecular Docking

Molecular docking was performed within the catalytic pocket site of the proteins using HTVS (high throughput virtual screening) and the standard precision (SP) mode of Grid using Glide [65,66] without applying any constraints. The prepared ligands were docked against grid-generated M^pro^ (PDB: 6LU7) and TMPRSS2 (PDB ID: 2OQ5) in the SP flexible mode [67]. The DockScore (DSore) representing the affinity of the docked ligands to enzymes was obtained from the project table file of the docked complexes.

#### 5.2.5. Induced Docking Fit (IDF)

IDF is an iterative combination of Glide (docking tool) and Prime (protein structure prediction and refinement). The docking with induced ligand–enzyme relaxation for accurate pose prediction was performed, where the selected ligands, as well as the binding sites, were free to move. Subsequent minimization of the highest-ranked pose was performed. The missing side chains and missing loops were filled using the Prime [68].

#### 5.2.6. Molecular Dynamics Simulation

Molecular dynamics (MD) simulation as a computational tool to monitor the stability and compatibility of the top-ranked ligand-enzyme complexes were performed using Desmond software [69]. The protein was prepared with filling the missing loop and side chains using Prime [68]. Then, the induced docked complex of the compound with protein was used as an initial conformation for MD simulation. The Desmond system builder was used to setup the MD system [70]. The orthorhombic box with periodic boundary conditions was generated with TIP4P solvent model including enzyme-ligand complexes as solute and the system was neutralized by adding suitable number of counter ions. The initial ligand–enzyme complex system was subject to an energy minimization. Simulation was carried out under NPT (constant number of atoms, constant pressure, and constant temperature) ensemble for 100 ns using the MD option of Desmond. Detailed information like protein and ligand root mean square deviation (RMSD) was calculated with respect to the initial frame backbone, root mean square fluctuation (RMSF), and ligand interaction profile were generated from the simulation trajectory of ligand–enzyme complexes. RMSD provides insights into the complex structural conformation throughout the MD simulation. The RMSF indicated the fluctuation along the protein chain.

#### 5.2.7. WaterMap Analysis

WaterMap analysis was employed to predict the profound impact of ligand structural variations on the binding affinity for the target protein [71]. The ligand displayed the hydration solvent that is occupying the binding pocket of the protein [72,73,74], and the WaterMap calculated the thermodynamics properties (dG, Tds, dH) of the hydration site in the binding pocket. The changes in the free energy resulting from displacing the water molecules in the active site can significantly affect the biological activity [75], which helps in understanding the molecular recognition pattern of the protein at the binding site. These were used to rationalize the SAR, drive the potency, and tune the selectivity [71]. WaterMap computed the hydration site properties (location, enthalpy, entropy, and free energy) through a combination of thermodynamic statistical analysis with molecular dynamics and solvent clustering. The MD simulation of the protein solvent without a ligand was run for 2 ns in order to determine the water molecules’ configuration at the binding site. The coordinates of the protein were restrained with 5.0 kcal/mol/Å2 harmonic potential applied to the initial positions of the heavy atoms, which ensures the convergence of the water sampling around the protein conformation. Water from the MD simulation were clustered to form localized hydration sites, and the thermodynamic properties of those sites were computed. The enthalpy was computed as the average nonbonded molecular mechanics interaction energies of the waters at the hydration site with the rest of the system [76]. The entropy was computed by numerically integrating a local expansion of spatial and orientation correlation functions, as described in the inhomogeneous solvation theory [77]. The relevant solvation thermodynamic quantities for the ligand were computed based on the amount of overlap with the hydration sites.

#### 5.2.8. PathFinder R-Group Enumeration

PathFinder is a computationally reaction-based enumeration tool that is used to generate a library of compounds using selected pathways via default or custom reagent libraries. Thus, it provides a facile and efficient tool for the rapid generation of a synthetically tractable library using practical available building blocks of all commercially available chemical reagents [78], and hence it can provide unique structural features required for improved potency, selectivity, and safety of a lead molecule. PathFinder was employed for R-group enumeration and retrosynthetic analysis of the starting hit **Compound 13**. Maximum depth was set as three to determine the synthetic steps that can be performed, and the maximum number of enumerated libraries were defined. The reaction was performed, while maintaining the core of the active hit and using all the possible reaction routes. In each route, the reagent that contained the core structure was kept, and the other reagents were varied one substitution at a time. The selection of the path that enables the generation of diverse enumerated structures was performed while maintaining the pharmacophoric scaffold.

#### 5.2.9. Ligand Designer

Ligand designer was used to enable relevant options, including a novel grow space to quickly identify the positions where ligand modifications should be. Further, it can be used for the visualization of 3D ligand–protein complexes for the optimum design and evaluation of ligand modifications. Ligand Designer, as an intuitive tool, demonstrated the ability to make a minor modification to the parent ligand in order to increase the potency of the compound. The lead optimization was evaluated with the visual inspection of 3D ligand–protein complex. The Ligand Designer tool analyzed the working places and reorganized the place for space growing. The pharmacophoric features determined the type of modification with favorable interaction for the stability of ligand–protein complex. Thus, it can provide an option for the examination and selection of the best enumerated structures based on multi-parameter optimization (MPO).

#### 5.2.10. R-Group Analysis

R-group mapping analysis was performed in Schrödinger suit. First, the input LigPrep structure with an IC_50_ value was converted to PIC_50_ values. The maximum common core was defined with Combi-Glide bond labeling and alignment of the structure for fingerprint similarity of the sidechain to minimize the number of attached R-groups. Heat map analysis displayed the effect of different functional group position with different color ranges, reflecting its pIC_50_ activity. A QSAR model was generated based on the pharmacophoric features, such as hydrogen bond donor (D), acceptor (A), hydrophobic group (H), negatively ionizable (N), positively ionizable (P), and aromatic ring (R).

### 5.3. Chemistry

^1^H NMR spectra were recorded on a Bruker spectrometer at 500 MHz. Chemical shifts were expressed in parts per million (ppm) relative to tetramethylsilane and the values of coupling constant (J) were represented in hertz (Hz). The signals were designated as follows: s, singlet; d, doublet; t, triplet; m, multiplet. Mass spectroscopic data were obtained through electrospray ionization (ESI) mass spectrometry.

**M3:** 1,2,5-Oxadiazole-3-carboximidic acid, 4,4′-(methylene-di-imino)-bis, bis[[(4-pyridyl)-methylene]-hydrazide. **^1^H NMR (DMSO-d6)** **δ:** 4.9 (t, 2H, CH_2_), 7.40 (d, 2H, J= 6.5, ArH), 7.5 (m, 5H, 2ArH, 3NH), 7.62 (t, 2H, NH), 8.40 (d, 3H, J= 8.5, ArH),8.66 (s, 2H, NH), 8.98 (d, 1H, ArH) (Supplementary Figure S1). MS analysis for C_19_H_18_N_14_O_2_: Calcd mass: 474.14, found (*m/z*, ES+): 475.26 (Supplementary Figure S2).

### 5.4. In Vitro Evaluation of Anti-SARS-CoV-2 Activity

The antiviral activity was carried out as previously described [12,79]. Briefly, 2.4 × 10^4^ Vero-E6 cells/well of 96-well tissue culture plates were incubated overnight in Dulbecco’s Modified Eagle’s Medium (DMEM) containing 10% fetal bovine serum (FBS) and 1% penicillin/streptomycin antibiotic mixture in a humidified 37 °C incubator under 5% CO_2_. The cell monolayers were subjected to SARS-CoV-2 (NRC-03-nhCoV strain, accession # EPI_ISL_430820) viral adsorption and further overlaid with 50 μL DMEM containing varying concentrations of the tested compounds. Following incubation at 37 °C and 5% CO_2_ for 72 h, the cells were fixed with 100 μL of 4% paraformaldehyde for 20 min and stained with 0.1% crystal violet in distilled water for 15 min at room temperature. The crystal violet dye was then dissolved in 100 μL methanol and the optical density of the obtained color was measured at 570 nm using Anthos Zenyth 200rt plate reader (Anthos Labtec Instruments, Heerhugowaard, Netherlands). The IC_50_ of the compound was measured using the formula below:

=[(OD test−OD blank)÷ (OD negative control−OD blank)]×100

### 5.5. Cell Viability Assay

The cytotoxic activity was performed using MTT (3-(4, 5-dimethylthiazolyl-2)-2, 5-diphenyltetrazolium bromide) assay according to Soliman et al. (2020) [80,81]. In 96-well plates, normal human dermal fibroblast cell line (HDF, 106-05A, Sigma, EU) were seeded at 4000 cells/100 μL and incubated with the tested compounds for 24 h at 37 °C, and 5% CO_2_. A 20 µL of sterile-filtered MTT reagent in PBS (5 mg/mL) was added to each well. The developed purple color following the addition of DMSO was measured using Multiskan Go machine (spectrophotometer) at 570 nm. Each experiment was repeated 6 times. The percentage cell viability was calculated following the following formula,

$$\begin{array}{ll} \mathrm{Cell} & \mathrm{viability}\;(\%)\\ & =\left[\left(OD\;test-OD\;blank\right)÷\;\left(OD\;negative\;control-OD\;blank\right)\right]\times 100 \end{array}$$

### 5.6. Main Protease (M^pro^) Assay

M^pro^ assay was carried out using 3CL Protease (3CL^pro^), Untagged (SARS-CoV-2) Assay Kit (CAT # 78042-1, BPS Bioscience, San Diego, CA, USA) according to the supplier’s protocols, following modifications as indicated in our previous publication [12]. The compound, in a total volume 2.5 µL, was incubated with 10 μL M^pro^ enzyme (1.5 ng/μL) in 384 black flat-bottom well plate. The reaction was performed in a reaction buffer made of 20 mM Tris-HCl pH 7.3, 100 mM NaCl, 1 mM EDTA, 0.01% BSA, and 1 mM 1,4-dithio-D, L-threitol (DTT) and the incubation was performed for 60 min at room temperature with slow shaking. Following incubation, 12.5 µL of 80 mM M^pro^ substrate (Dabcyl-KTSAVLQSGFRKME-Edans fluorogenic substrate) was added in dark and allowed to incubate for 1 h at room temperature. The fluorescence intensity was measured by a microtiter plate-reader (Synergy H1, Biotek Ltd., Winooski, VT, USA) at emission and excitation wavelengths of 460 and 360 nm, respectively. The inhibition activity of the compound was evaluated at different concentrations (0.003, 0.007, 0.013, 0.026, 0.053, 0.1054, and 0.211 µM), while cysteine protease covalent inhibitor (GC376) was employed as positive control at a concentration 100 µM according to Fu et al. (2020) [82], and the reaction without inhibitor and enzymes was employed as negative control. The inhibitory activity was plotted against the logarithm of inhibitor concentrations to calculate the IC_50_.

### 5.7. Furin Protease Assay

Furin protease assay was performed using a furin protease assay kit (Cat # 78040, BPS Bioscience, San Diego, CA, USA) according to the manufacturer’s instructions, following modifications indicated in our previous publication [12]. Initially, 10 µL of the compound at different concentrations (0.007, 0.013, 0.026, 0.053, and 0.1054 µM) was incubated with 50 μL recombinant furin enzyme at 0.5 ng/µL for 30 min at 37 °C in 96 black flat-bottom well plate. Furin protease substrate (40 µL) was then added, and the relative fluorescence value was measured after 1 h with excitation and emission wavelengths 380 and 460 nm, respectively. For positive control, chloro-methyl-ketone at 0.5 µM was employed according to Hoffman et al. (2020) [83], while the reaction without inhibitor was used as negative control.

### 5.8. TMPRSS2 Fluorogenic Assay

TMPRSS2 fluorogenic assay kit (CAT # 78083, BPS Bioscience, San Diego, CA, USA) was used to evaluate the inhibition activity of the compounds on TMPRSS2 enzyme following the supplier protocol and according to our previous publication [12]. Briefly, 30 µL TMPRSS2 (5 ng/µL) was added to 10 µL of compound at different concentrations (0.007, 0.013, 0.026, 0.053, and 0.1054 µM). Following incubation for 30 min at room temperature, 10 µL of TMPRSS2 substrate (50 µM) was added, and the fluorescence intensity was measured in dark by a microtiter plate-reader (Synergy H1, Biotek Ltd., Winooski, VT, USA) at an emission and excitation wavelengths 383 and 455 nm, respectively. Camostat mesylate (10 µM) was used as a positive control according to Hoffmann et al. (2020) [84], while reaction without inhibitor and enzyme was used as negative control.

### 5.9. Preparation of Compound-Loaded ZnO Nanoparticles

Loading the compound on ZnO NPs was performed according to Wang et al. (2017) with modifications [85]. Briefly, 25 mg ZnO NPs (99+%, 10–30 nm, US Research Nanomaterials, Inc, Houston, TX, USA) were dispersed in 5 mL distilled water by ultrasonic sonicator bath (Branson, St. Louis, MO, USA) for 5 min, then 3.16 µM of the compound that was dissolved in DMSO was added. The mixture was then stirred for 36 h followed by centrifugation for 20 min at 13,000 rpm. The produced compound-loaded ZnO nanoparticles were washed three times with water and then freeze-dried overnight using a benchtop freeze dryer (Labconco, MO, USA). The amount of compound adsorbed on ZnO NPs was determined by incubating the compound–ZnO NPs with DMSO for 30 min followed by centrifugation for 20 min at 13,000 rpm. The adsorbed amount of the compound was extracted from the compound–ZnO NPs and measured at 330 nm using UV/VIS spectrophotometer (SYNERGY H1, Biotek Ltd., Winooski, VT, USA).

The drug entrapment efficiency (EE) was calculated as follows:

Entrapment efficiency % = (Amount of drug adsorbed/Total amount of drug used) × 100

### 5.10. Characterization of Compound-ZnO Nanoparticles

The zeta-potential of the compound–ZnO NPs were evaluated using Zetasizer (Malvern, Cambridge, UK). NPs were diluted with distilled water and sonicated for 1 h in an ultrasonic sonicator bath (Branson, St. Louis, MO, USA), then the laser doppler velocimetry (LDV) technique was employed to measure the zeta potential (mV). Further, the morphological characteristics of the formed compound–ZnO NPs were examined by a scanning electron microscope (JSM-633OF; JEOL Ltd., Tokyo, Japan). Compound–ZnO NPs samples were initially suspended in distilled water, and then one drop was placed on a clean slide cover and left to dry at room temperature. The dried sample was mounted on carbon tape and sputter-coated with gold. The samples coated with gold were then scanned and photomicrographs were taken at an acceleration voltage of 15 kV. The cell viability and in vitro activity of ZnO NPs and compound–ZnO NPs against SARS-CoV-2 were assessed as mentioned earlier.

### 5.11. Statistical Analysis

The data were obtained and graphed using GraphPad Prism (8.01, GraphPad Inc., La Jolla, CA, USA). The enzyme inhibition and cytotoxic activities of the compounds were analyzed using one-way analysis of variance (ANOVA) using Bonferroni’s multiple comparisons test. *p* < 0.05 was considered as significant. The data display the mean ± SEM of 3–6 replicas.

## Figures and Tables

**Table 1:** Docking score of shortlisted compounds against M^pro^ and TMPRSS2.

Compound	Docking Score with	
	M^PRO^	TMPRSS2
**M1**	−9.97	−8.11
**M2**	−8.16	−8.53
**M3**	−8.79	−9.81
**M4**	−9.67	−9.32
**M5**	−7.37	−6.32
**Compound 13**	−7.01	−9.86

**Table 2:** Molecular modeling of the shortlisted compounds (**M1**–**M5**) within the binding active site of SARS-CoV-2 M^pro^ when compared to **Compound 13**.

Compound	Moiety	Interaction	Amino Acid Residue
**M1**	NH N of oxadiazole Phenyl ring	H-bond H-bond pi-pi stacking bond Hydrophobic bond	Glu-166 Asn-142 His-41 His-41, Met-49, Cys-145, Met-165 and Pro-168
**M2**	NH_2_	2H-bonds Hydrophobic bond	Phe-140 and Glu-166 His-41, Met-49, Cys-145, Met-165 and Gln-189
**M3**	NH_2_ NH N pyridyl moiety and CH	2H-bonds H-bond H-bond Hydrophobic bond	Cys-44 and Thr-25 Asn-142 Glu-166 Met-49, Cys-44, Glu-166 and Met-165
**M4**	NH_2_ Cl	2- H-bonds Halogen bond Hydrophobic bond	Glu-166 and Phe-140 Gln-192 Met-49, Glu-166, Cys-145, Met-165, Gln-189, Gln-192 and Pro-168
**M5**	NH_2_	2H-bonds Hydrophobic bond	Glu-166 and Phe-140 Cys-44, His-41, Met-49, Gln-189 and Pro-168
**Compound 13**	Phenolic OH NH_2_ NH_2_ N Phenolic OH Phenyl ring and CH	H-bond H-bond H-bond pi-pi stacking bond Hydrophobic bond	Glu-166 Phe-140 Thr-26 His-40 Met-44, Cys-44 and Met-165.

**Table 3:** Molecular modeling of the shortlisted compounds (**M1**–**M5**) within the binding active site of human TMPRSS2 when compared to **Compound 13**.

Compound	Moiety	Interaction	Amino Acid Residue
**M1**	NH N	H-bond H-bond Hydrophobic bond	Gln-192 Ser-195 Arg-41, Cys-219 and Tyr-228
**M2**	NH_2_ NH	H-bond H-bond Hydrophobic bond	Gln-192 Ser-214 Tyr-36, Arg-41, Gln-192, Val-213 and Cys-219
**M3**	2-NH N of 4-pyridyl moiety N pyridinyl moiety and CH	2H-bonds H-bond H-bond Hydrophobic bond	Ser-214 Arg-41 Glu-218 His-57, Arg-41 and Cys-219 and Cys-42
**M4**	NH_2_ Cl N	3-H-bonds 3-Halogen bond H-bond Hydrophobic bond	His-57, Ser-214, Gln-192 Ala-220, Ser-214, Ser-39 Arg-41 Arg-41, Val-213, Cys-219 and Tyr-228
**M5**	OCH3 NH	H-bond H-bond Hydrophobic bond	Arg-41 Gln-192 Arg-41, Tyr-149, Ala-190, Val-213 and Cys-219
**Compound 13**	Phenolic OH NH_2_ NH_2_ N Phenolic OH Phenyl ring and CH	H-bond H-bond H-bond H-bond H-bond Hydrophobic bond	His-40 Gln-192 Ser-214 Arg-41 Glu-218 Arg-41, Ala-190, Val-213, Cys-42 and Trp-36

## Supplementary Materials

The following are available online at https://www.mdpi.com/article/10.3390/ijms22169057/s1.

## Abbreviations

**A;** hydrogen bond acceptor, **3CL^pro^;** 3CL-Protease, **BSA**; bovine serum albumin, **CC_50_**; 50% cytotoxic concentration, **CDC;** Center for Disease Control and Prevention, **COVID-19**; coronavirus disease-2019, **dG;** differential Gibbs free energy, **DMEM;** Dulbecco’s Modified Eagle’s Medium, **DMSO**; dimethyl sulfoxide, **DTT;** dithiothreitol, **EDTA;** ethylene diamine tetra-acetic acid, **EE**; entrapment efficiency, **EUA;** emergency use authorization, **FDA**; Food and Drug Administration, **FBS;** fetal bovine serum, **H**; hydrophobic group, **H-bond;** hydrogen bond, **HBA;** hydrogen bond acceptor, **HDF;** human dermal fibroblast, **HIV;** human immunodeficiency virus, **H1N1**; influenza virus, **HTVS;** high throughput virtual screening, **IC_50_**; 50% inhibitory concentration, **Ligprep;** ligand preparation, **LDV;** laser doppler velocimetry, **MD**; molecular dynamics, **M^pro^;** main protease, **MTT**; 3-(4,5-dimethylthiazol-2-yl)-2,5 diphenyl tetrazolium bromide, **N;** negatively ionizable, **NPT;** constant number of atoms, constant pressure, and constant temperature, **P**; positively ionizable, **PAINS;** pan-assay interference compounds, **PBS;** phosphate buffered saline, **PDB**; protein data bank, **PLpro**; papain-like protease, **QSAR;** quantitative structure–activity relationship, **RCSB;** Research Collaboratory for Structural Bioinformatics, **R;** aromatic ring, **RdRp**; RNA-dependent RNA polymerase, **ROS;** reactive oxygen species, **RMSD;** Root mean square deviation, **RMSF**; Root mean square fluctuation, **SAR;** structure–activity relationships, **SARS-CoV-2;** severe acute respiratory syndrome coronavirus 2, **SI;** selectivity index, **SP**; standard precision, **TMPRSS2**; human transmembrane serine protease 2, **WHO;** World Health Organization, **ZnO NPs;** zinc oxide nanoparticles.
