# Supplementary material for: Iterated Virtual Screening-Assisted Antiviral and Enzyme Inhibition Assays Reveal the Discovery of Novel Promising Anti-SARS-CoV-2 with Dual Activity
Source: Int J Mol Sci. 2021 Aug 22;22(16):9057. doi: 10.3390/ijms22169057 (PMC8396542; doi:10.3390/ijms22169057)
Supplement: Supplementary file 1 [file ijms-22-09057-s001.zip › ijms-1325532-supplementary.pdf]

Supplementary Data

Supplementary Table 1. Molecular modeling of the 20 selected compounds within the binding active site of SARS-CoV-2 M^{pro}.

Compound	Moiety	Interaction	Amino acid residue
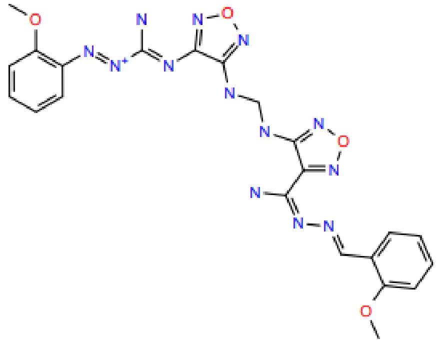	NH2	2H-bonds<br>Hydrophobic bond	Glu-166 and Phe-140<br>Cys-44, Hie-41, Met-49, Gln-189 and Pro-168
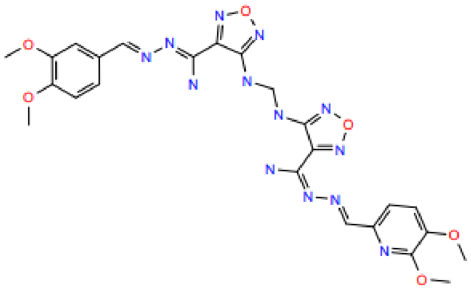	NH<br>N of oxadiazole<br>Phenyl ring	H-bond<br>H-bond<br>pi-pi stacking bond<br>Hydrophobic bond	Glu-166<br>Asn-142<br>Hie-41<br>Hie-41, Met-49, Cys-145, Met-165 and Pro-168
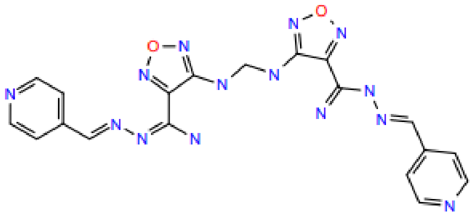	NH2<br>NH<br>N<br>pyridyl moiety and CH	2H-bonds<br>H-bond<br>H-bond<br>Hydrophobic bond	Cys-44 and Thr-25<br>Asn-142<br>Glu-166<br>Met-49, Cys-44, Glu-166 and Met-165
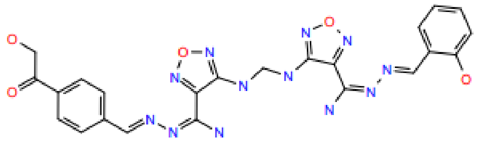	NH2<br>NH<br>OH<br>Phenyl ring and oxadiazole ring	H-bond<br>H-bond<br>H-bond<br>Hydrophobic bond	Phe-140<br>Cys-145<br>His-164<br>Hie-41
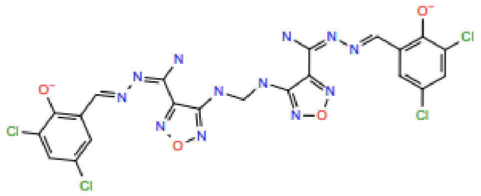	NH2	2H-bonds<br>Hydrophobic bond	Phe-140 and Glu-166<br>Hie-41, Met-49, Cys-145, Met-165 and Gln-189

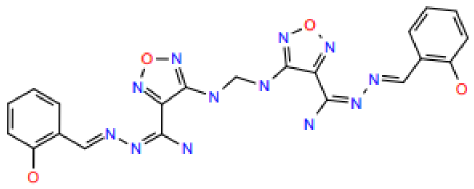	Phenolic OH<br>NH2<br>NH2<br>N<br>Phenolic OH<br>Phenyl ring and CH	H-bond<br>H-bond<br>H-bond<br>pi-pi stacking bond<br>Hydrophobic bond	Glu-166<br>Phe-140<br>Thr-26<br>His-40<br>Met-44, Cys-44 and Met-165.
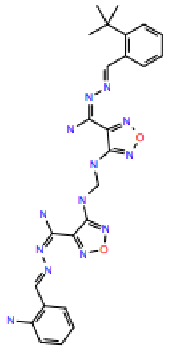	2NH<br>NH2	H-bonds<br>H-bond	Asn-142<br>Leu-141 and Glu-166
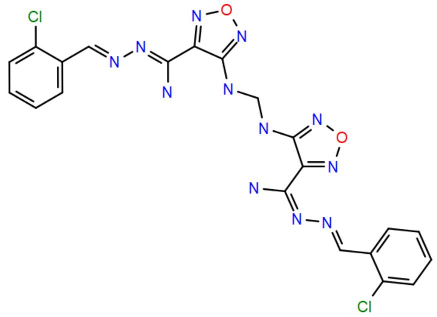	NH2<br>Phenyl ring and CH	2H-bonds<br>Hydrophobic bond	Phe-140 and Glu-166<br>Hie-41, Met-49, Cys-145, Met-165 and Gln-189
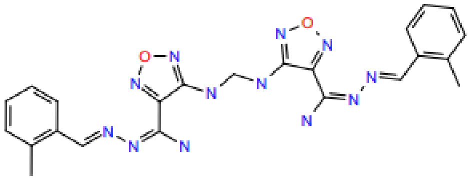	NH2<br>Phenyl ring and CH	2H-bonds<br>Hydrophobic bond	Glu-166<br>Cys-145.
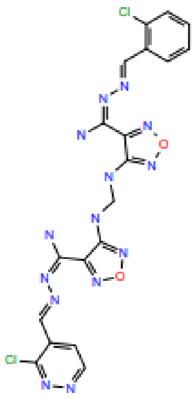	NH2<br>Cl<br>Phenyl ring and CH	H-bond<br>Halogen bond<br>Hydrophobic bond	His-164<br>Ser-144<br>Gln-189

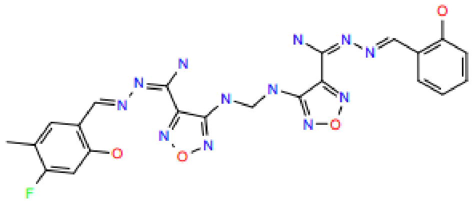	Phenolic OH<br>NH<br>N of oxadiazoles<br>Phenyl ring and CH	H-bond<br>H-bond<br>H-bond<br>Hydrophobic bond	His-164<br>Asn-142<br>Cys-145<br>Hie-41
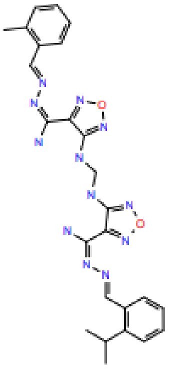	NH<br>oxadiazoles<br>Phenyl ring<br>Isopropyl ring	H-bond<br>Hydrophobic bond<br>Hydrophobic bond	Asn-142<br>Hie-41<br>Met-49
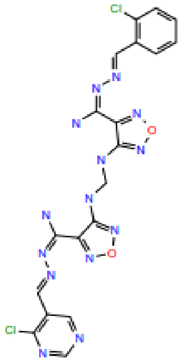	N<br>oxadiazole and ring	H-bond<br>2-Hydrophobic bonds	Glu-166<br>Asn-142<br>Hie-41
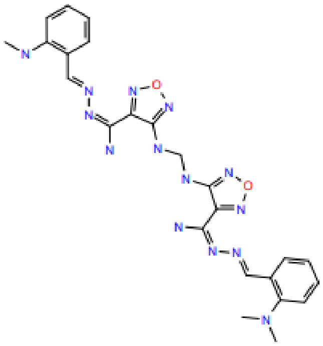	O of oxadiazole<br>NH2<br>Phenyl ring	H-bond<br>H-bond<br>2-Hydrophobic bonds	Gly-143<br>Leu-141<br>Hie-41

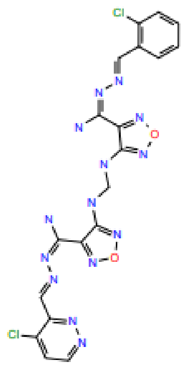	NH2<br>Cl<br>Phenyl ring and CH	H-bond<br>Halogen bond<br>Hydrophobic bond	Glu-166<br>Cys-145 and Gly-143<br>Gln-189
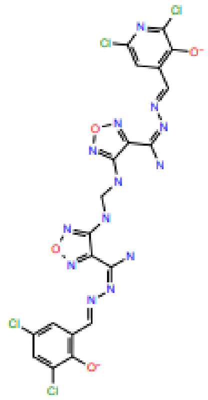	NH2<br>OH<br>Cl	H-bond<br>H-bond<br>Halogen bond	Glu-166<br>Cys-145<br>Ala-191
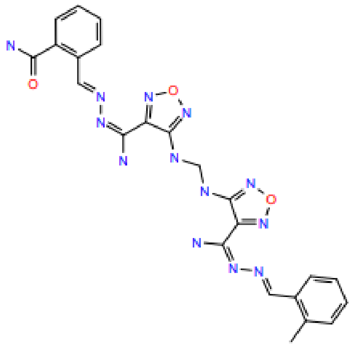	NH<br>NH2<br>Oxadiazole	H-bonds<br>H-bond<br>Hydrophobic bond	Glu-166 and Gln-189<br>His-164<br>Hie-41
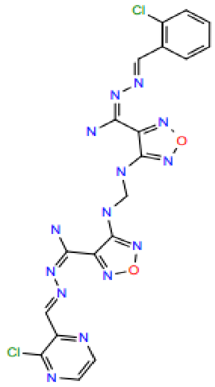	NH2<br>NH2<br>Ring	H-bond<br>H-bond<br>Hydrophobic bond	His-164<br>Gln-189<br>Gln-192

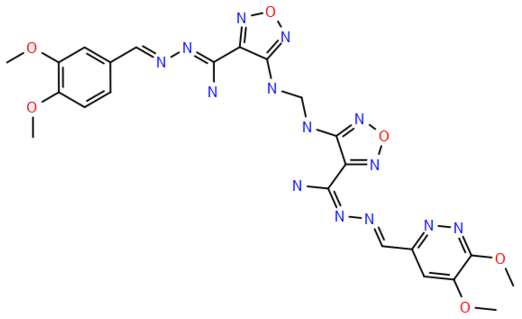	NH2<br>Pyridazine ring<br>Phenyl ring	H-bond<br>H-bond<br>Hydrophobic bond	Glu-166<br>Gly-143<br>Pro-168
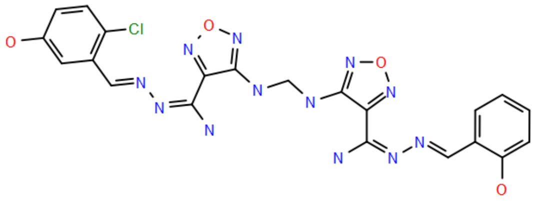	NH<br>OH<br>NH2<br>Oxadiazole	H-bonds<br>H-bond<br>H-bond<br>Hydrophobic bond	Asn-142<br>His-164 and Thr-190<br>Phe-140<br>Hie-41

Supplementary Table 2. Molecular modeling of the 20 selected compounds within the binding active site of SARS-CoV-2 TMPRSS2.

Compound	Moiety	Interaction	Amino acid residue
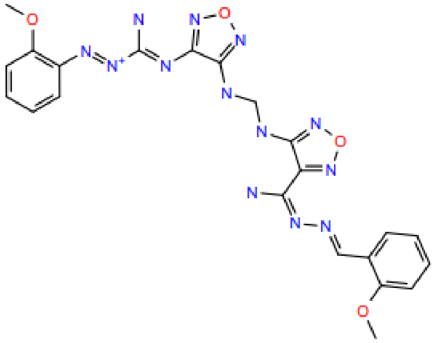	OCH3<br>NH	H-bond<br>H-bond<br>Hydrophobic bond	Arg-41<br>Gln-192<br>Arg-41, Tyr-149, Ala-190, Val-213 and Cys-219
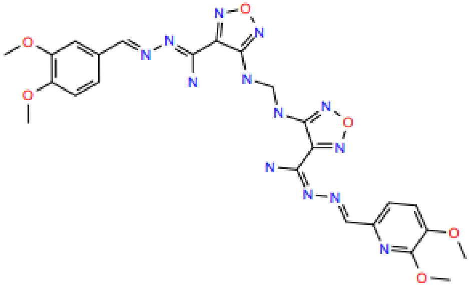	NH<br>N	H-bond<br>H-bond<br>Hydrophobic bond	Gln-192<br>Ser-195<br>Arg-41, Cys-219 and Tyr-228
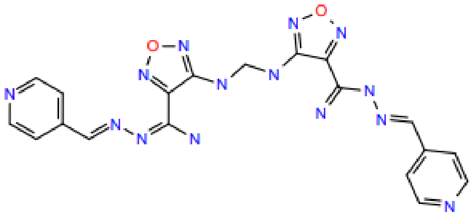	2-NH<br>N of 4-pyridyl moiety<br>N<br>pyridinyl moiety and CH	2H-bonds<br>H-bond<br>H-bond<br>Hydrophobic bond	Ser-214<br>Arg-41<br>Glu-218<br><br>His-57, Arg-41 and Cys-219 and Cys-42
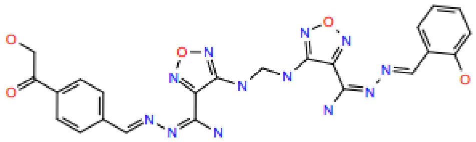	NH2<br>N of oxadiazole<br>Phenolic OH<br>OH	H-bond<br>H-bond<br>H-bond<br>H-bond	His-57<br>Thr-26<br>Gly-148 and Gly-216<br>Gly-148
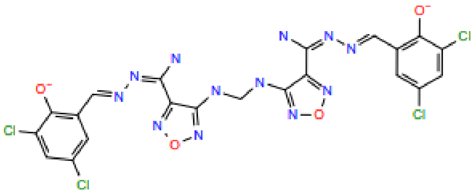	NH2<br>Cl<br>N	3- H-bonds<br>3- Halogen bond<br>H-bond<br>Hydrophobic bond	His-57, Ser-214, Gln-192<br>Ala-220, Ser-214, Ser-39<br>Arg-41<br>Arg-41, Val-213, Cys-219 and Tyr-228

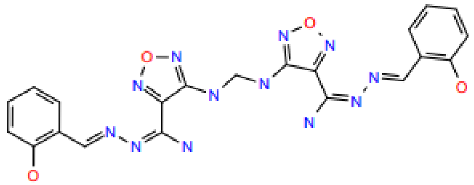	Phenolic OH<br>NH2<br>NH2<br>N<br>Phenolic OH<br>Phenyl ring and CH	H-bond<br>H-bond<br>H-bond<br>H-bond<br>H-bond<br>Hydrophobic bond	Hie-40<br>Gln-192<br>Ser-214<br>Arg-41<br>Glu-218<br>Arg-41, Ala-190, Val-213, Cys-42 and Trp-36
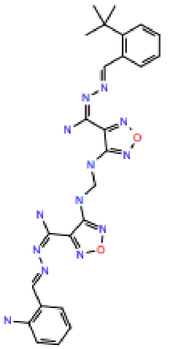	NH2<br>N<br>Oxadiazole ring	H-bond<br>H-bonds<br>Hydrophobic bond	Ser-195<br>Gln-192<br>His-57
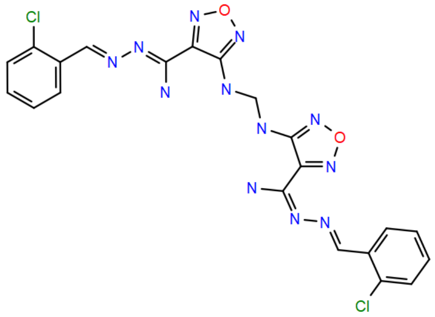	NH2<br>NH	H-bond<br>H-bond<br>Hydrophobic bond	Gln-192<br>Ser-214<br>Tyr-36, Arg-41, Gln-192, Val-213 and Cys-219
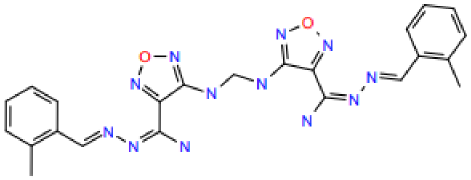	NH2<br>Phenyl ring	H-bonds<br>Hydrophobic bond	Thr-62<br>His-57 and Ala-190
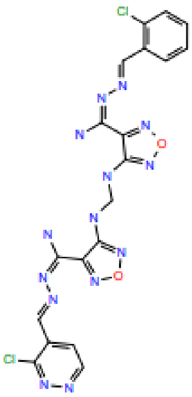	NH2<br>Phenyl ring	H-bonds<br>Hydrophobic bond	Gly-216<br>Ala-190

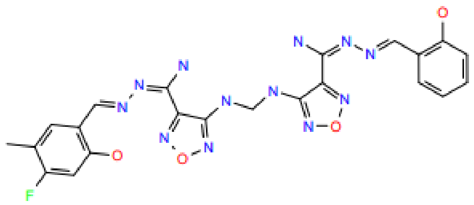	Phenolic OH<br>NH2<br>N<br>Oxadiazole	H-bond<br>H-bond<br>H-bond<br>Hydrophobic bond	Glu-218 and Gly-216<br>Pro-97<br>Hie-99<br>Hie-96
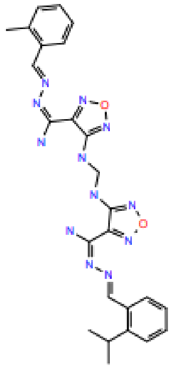	NH<br>Oxadiazole<br>Phenyl ring	H-bond<br>Hydrophobic bond<br>Hydrophobic bond	Ser-195<br>His-57<br>Gln-192
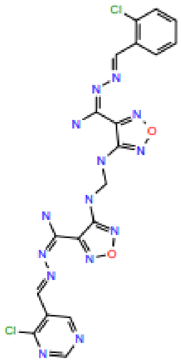	N<br>oxadiazole and phenyl ring	H-bond<br>2-Hydrophobic bonds	Gly-216<br>Tyr-149<br>Gln-192
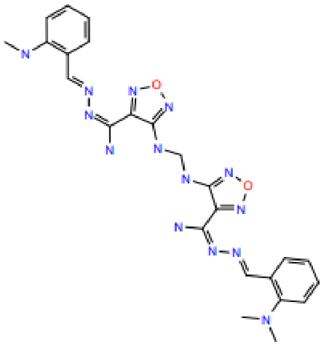	N of oxadiazole<br>N<br>Phenyl ring	H-bond<br>H-bond<br>Hydrophobic bonds	Gln-192<br>Hie-99<br>Hie-99

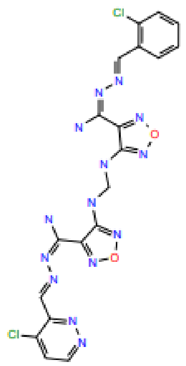	NH2<br>Cl<br>NH<br>Oxadiazole	H-bond<br>Halogen bond<br>H-bond<br>Hydrophobic bond	Thr-62 and Cys-58<br>Arg-41<br>His-57<br>Hie-96
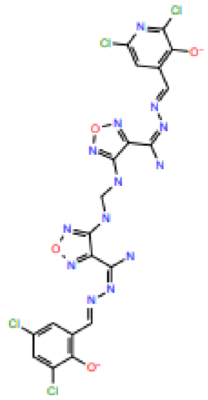	NH2<br>N<br>Pyridine	H-bond<br>H-bond<br>Hydrophobic bond	Glu-218 and Asp-147<br>Gly-218<br>Hie-99
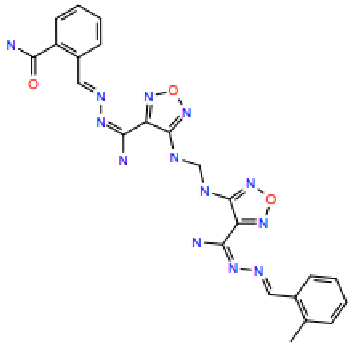	NH<br>NH2<br>N	H-bonds<br>H-bond<br>H-bond	His-57<br>Arg-41<br>Gln-192
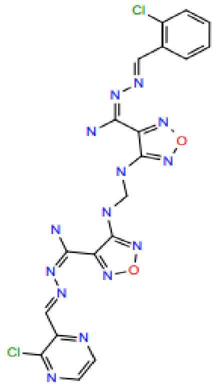	N<br>Cl<br>N of pyrazine	H-bond<br>Halogen bond<br>H-bond	Gly-193 and Hie-99<br>Glu-218<br>Gln-192

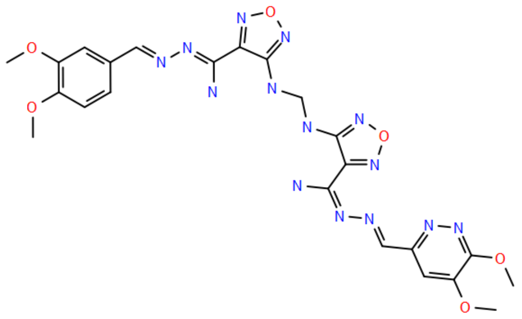	NH2<br>NH	H-bond<br>H-bond	Gln-192<br>Gly-218
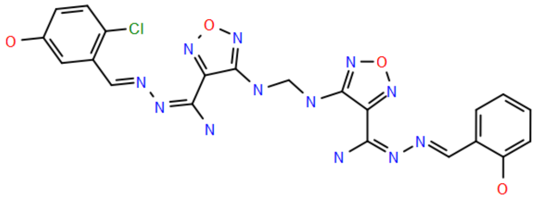	N<br>OH<br>NH2<br>Oxadiazole	H-bond<br>2H-bond<br>H-bond<br>Hydrophobic bond	Hie-99<br>Gly-216 and Glu-218<br>Pro-97<br>Hie-96

Supplementary Table 3. WaterMap analysis of M^{pro} and TMPRSS2.

Site	Free energy (dG) of TMPRSS2	Free energy (dG) of M^{pro}	Site	Free energy (dG) of TMPRSS2	Free energy (dG) of M^{pro}
1	2.92	6.94	55	1.31	2.45
2	8.1	2.73	56	-0.44	-0.89
3	7.39	6.51	57	2.12	3.17
4	3.49	7.4	58	0.9	1.88
5	4.23	2.27	59	1.04	2.08
6	6.66	1.89	60	1.58	6.28
7	6.21	2.39	61	1.54	1.46
8	5.05	3.34	62	1.49	2.81
9	4.71	2.2	63	0.49	2.19
10	2.43	7.52	64	2	3.16
11	6.01	1.77	65	2.22	1.88
12	3.35	1.79	66	1.86	-0.5
13	3.82	1.3	67	3.83	2.56
14	4.45	2.41	68	2.26	1.21
15	3.5	1.6	69	3.57	0.98
16	2.91	2.55	70	0.71	2.03
17	2.44	2.22	71	1.54	1.41
18	3.25	3.19	72	1.57	1.51
19	3.21	0.92	73	-1.72	2.05
20	1.12	-0.02	74	1.84	1.3
21	2.91	1.42	75	1.66	1.04
22	1.04	4.02	76	2.77	1.01
23	2.88	2.87	77	0.61	1.49
24	1.4	4.15	78	1.9	1.17
25	-0.27	-0.28	79	1.02	1.67
26	3.13	1.82	80	1.45	1.58
27	4.21	1.24	81	0.69	1.28
28	1.39	3.76	82	-0.17	1.73
29	6.58	2.25	83	0.89	2.48
30	1.14	3.89	84	1.7	1.02
31	1.75	3.8	85	2.64	0.79
32	2.38	2.34	86	0.72	0.09
33	-1.38	2.36	87	2.74	0.79
34	1.36	4.06	88	1.23	2.45
35	2.74	1.87	89	0.65	1.06
36	0.87	1.94	90	2.08	1.35
37	0.85	3.18	91	1.66	0.6
38	1.53	2.23	92	-0.79	2.02
39	5.3	1.73	93	1.77	1.85
40	1.96	2.42	94	0.42	1.34
41	-0.4	0.72	95	0.57	1.16
42	1.42	3.65	96	1.52	0.82
43	0.12	1.19	97	2.24	1.35
44	1.68	2.93	98	2.22	1.57
45	-0.76	2.41	99	2.03	2.27
46	2.15	2.3	100	3.17	1.44
47	2.75	3.15	101	1.84	1.3
48	2.53	2.34	102	0.55	2.49
49	1.06	0.15	103	-1.15	0.79
50	1.47	1.41	104	1.9	1.48
51	1.44	1.19	105	2.42	0.78
52	-0.29	3.43	106		0.92
53	3.89	4.4	107		2.19
54	2.6	1.88	108		1.2
			109		1.57
			110		1.2
			111		1.52

Supplementary Figures

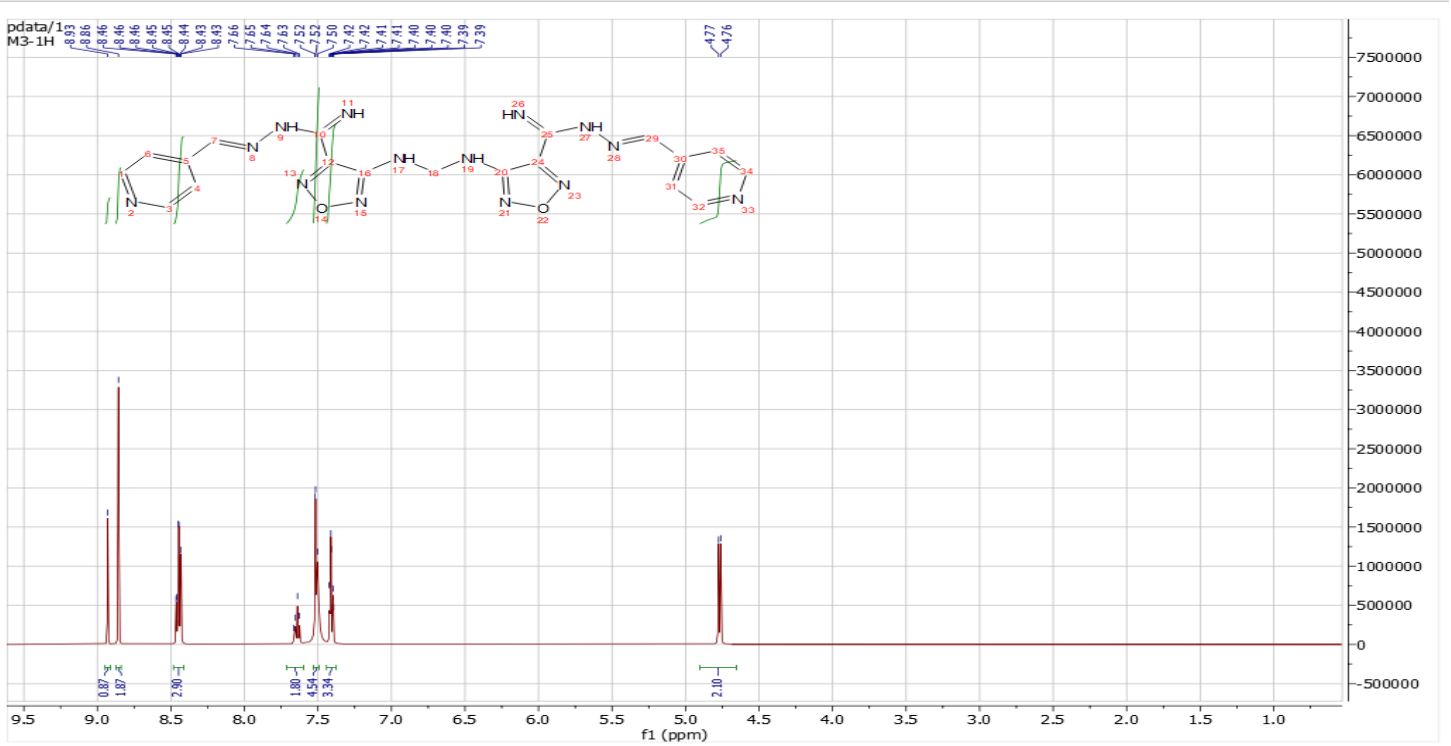

Supplementary Figure 1. NMR data of compound M3.

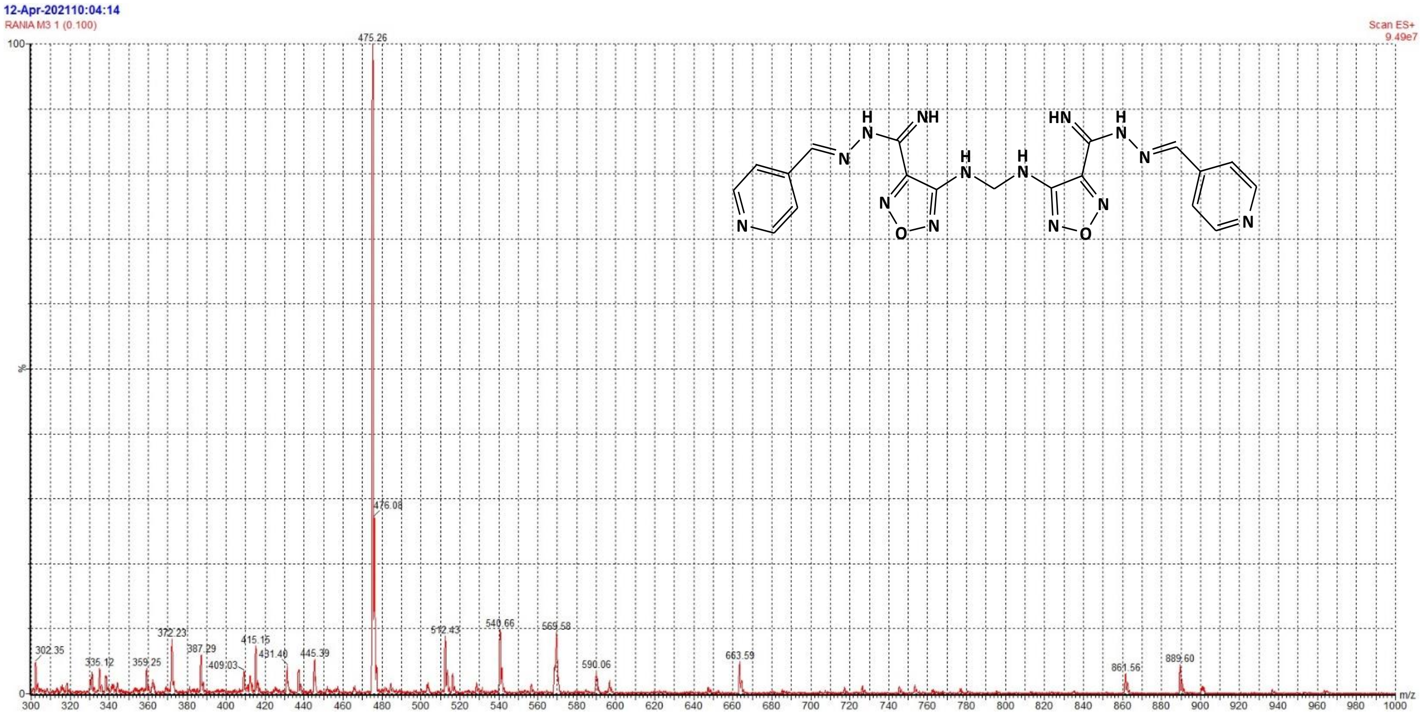

Supplementary Figure 2. Mass spectrometry of compound M3.

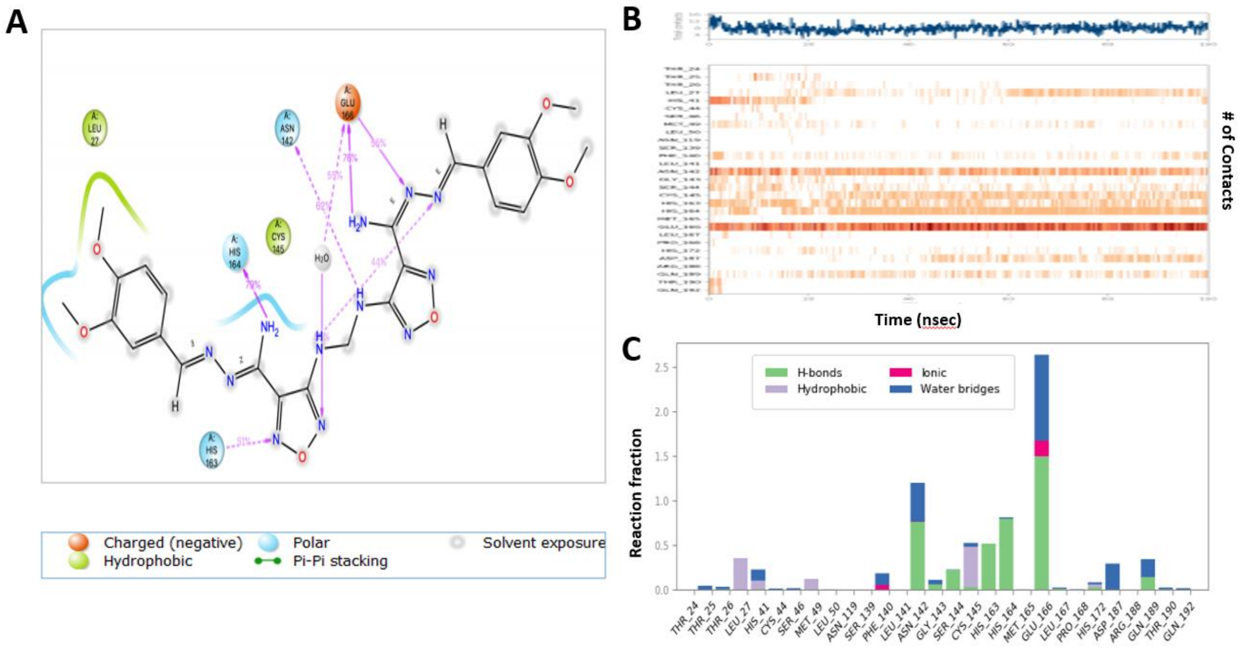

Supplementary Figure 3. Interaction diagram of M1 with M^{pro} protein observed during the molecular dynamic simulation. **(A)** Protein-ligand interaction diagram**.** **(B)** The top panel showed the specific contact of M^{pro} protein with **M1** in each trajectory course, the bottom panel showed the amino acid residues that interact with the ligand in the trajectory time frame. The residues making more than one contact were shown in darker color shade. **(C)** Schematic diagram of ligand interaction with the amino acid residues of protein during MD simulation.

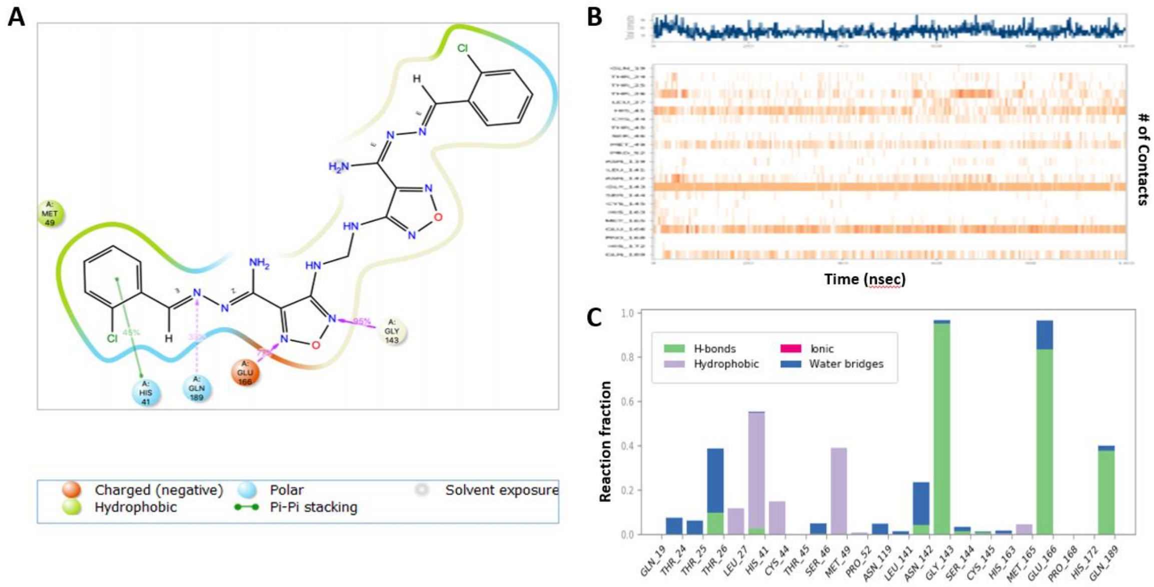

Supplementary Figure 4. Interaction diagram of M2 with M^{pro} protein observed during the molecular dynamic simulation. **(A)** Protein-ligand interaction diagram**.** **(B)** The top panel showed the specific contact of M^{pro} protein with **M2** in each trajectory course, the bottom panel showed the amino acid residues that interact with the ligand in the trajectory time frame. The residues making more than one contact were shown in darker color shade. **(C)** Schematic diagram of ligand interaction with the amino acid residues of protein during MD simulation.

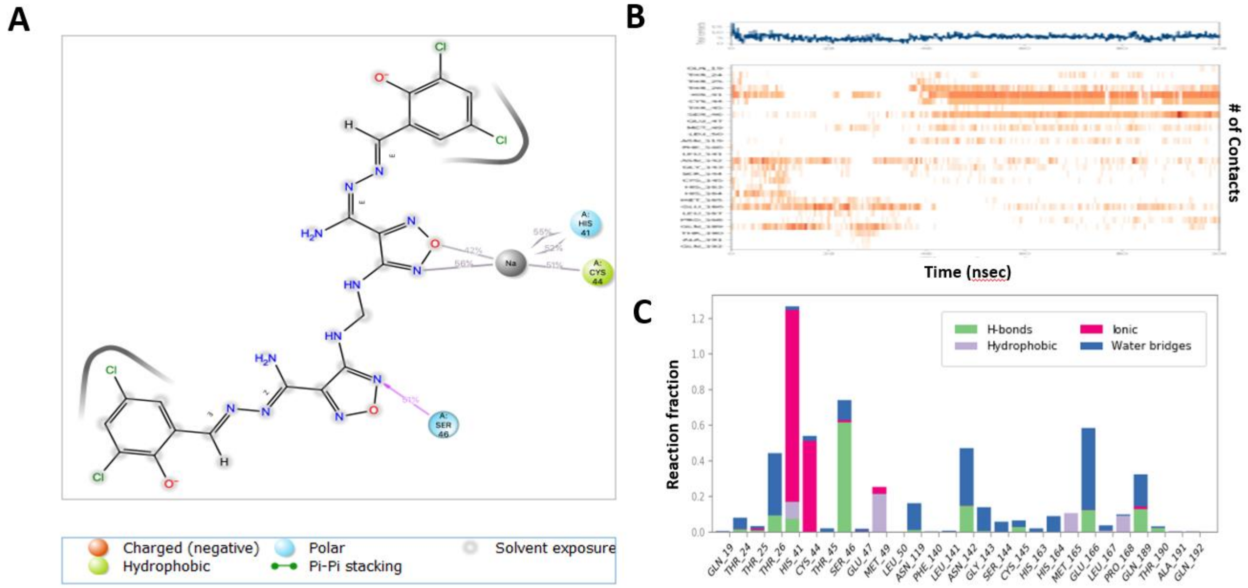

Supplementary Figure 5. Interaction diagram of M4 with M^{pro} protein observed during the molecular dynamic simulation. (A) Protein-ligand interaction diagram**. (B)** The top panel showed the specific contact of M^{pro} protein with **M4** in each trajectory course, the bottom panel showed the amino acid residues that interact with the ligand in the trajectory time frame. The residues making more than one contact were shown in darker color shade. **(C)** Schematic diagram of ligand interaction with the amino acid residues of protein during MD simulation.

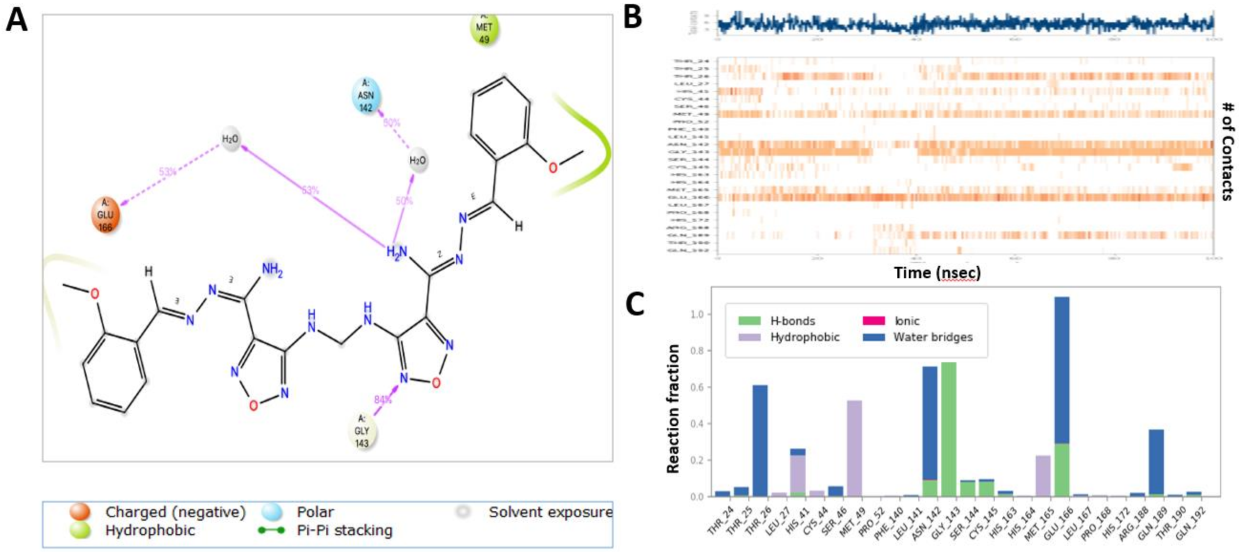

Supplementary Figure 6. Interaction diagram of M5 with M^{pro} protein observed during the molecular dynamic simulation. (A) Protein-ligand interaction diagram**. (B)** The top panel showed the specific contact of M^{pro} protein with **M5** in each trajectory course, the bottom panel showed the amino acid residues that interact with the ligand in the trajectory time frame. The residues making more than one contact were shown in darker color shade. **(C)** Schematic diagram of ligand interaction with the amino acid residues of protein during MD simulation.

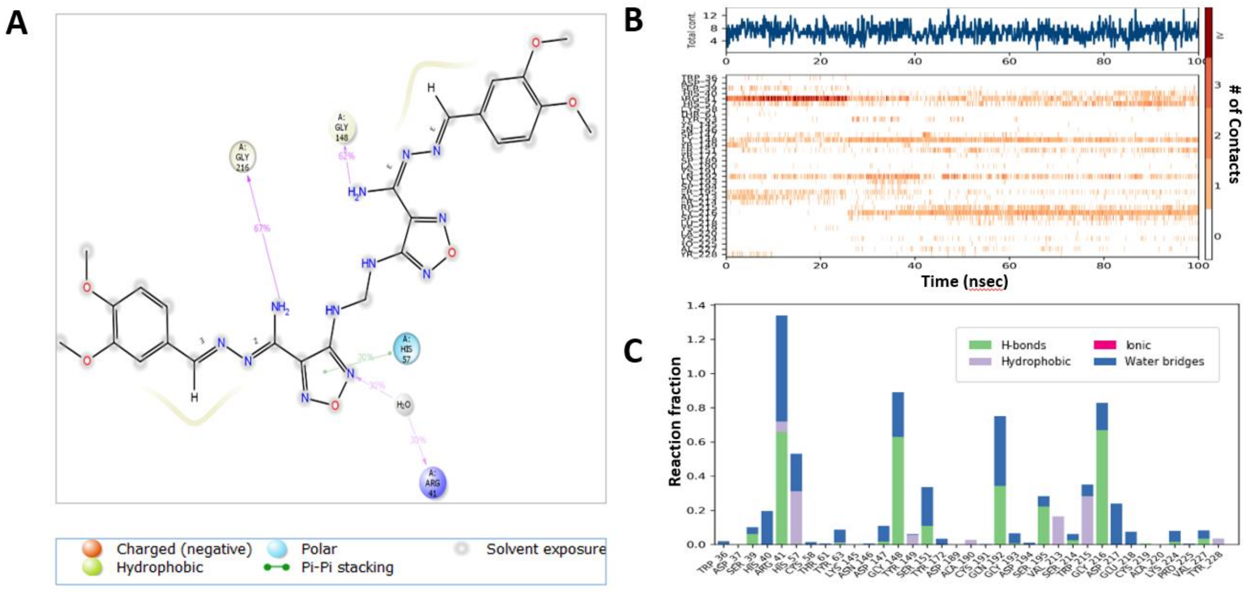

Supplementary Figure 7. Interaction diagram of M1 with TMPRSS2 protein observed during the molecular dynamic simulation. (A) Protein-ligand interaction diagram**. (B)** The top panel showed the specific contact of TMPRSS2 protein with **M1** in each trajectory course, the bottom panel showed the amino acid residues that interact with the ligand in the trajectory time frame. The residues making more than one contact were shown in darker color shade. **(C)** Schematic diagram of ligand interaction with the amino acid residues of protein during MD simulation.

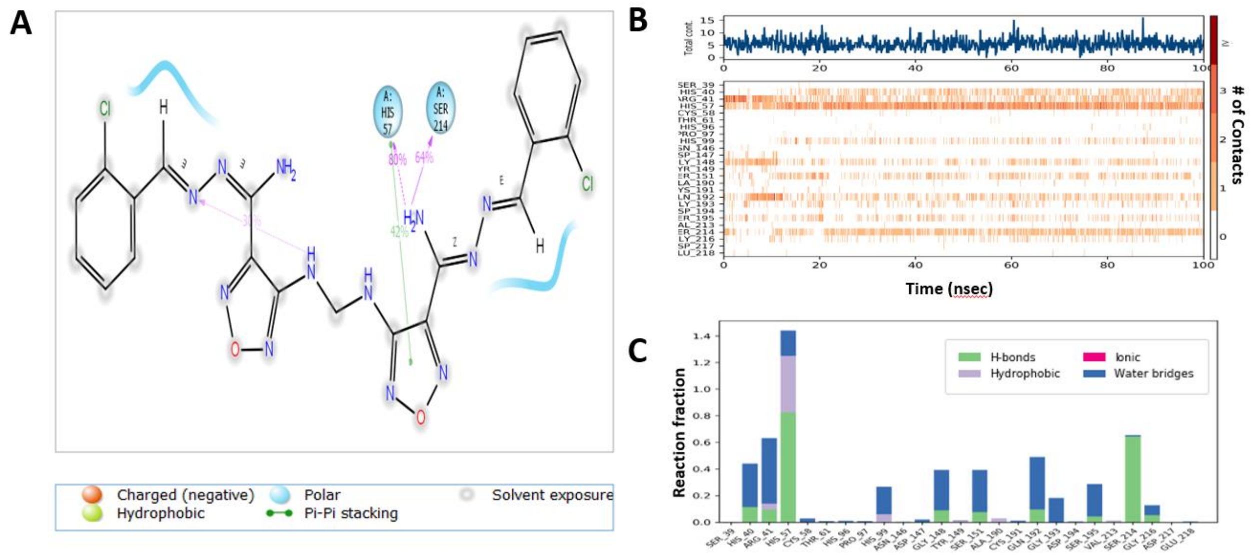

Supplementary Figure 8. Interaction diagram of M2 with TMPRSS2 protein observed during the molecular dynamic simulation. (A) Protein-ligand interaction diagram**. (B)** The top panel showed the specific contact of TMPRSS2 protein with **M2** in each trajectory course, the bottom panel showed the amino acid residues that interact with the ligand in the trajectory time frame. The residues making more than one contact were shown in darker color shade. **(C)** Schematic diagram of ligand interaction with the amino acid residues of protein during MD simulation.

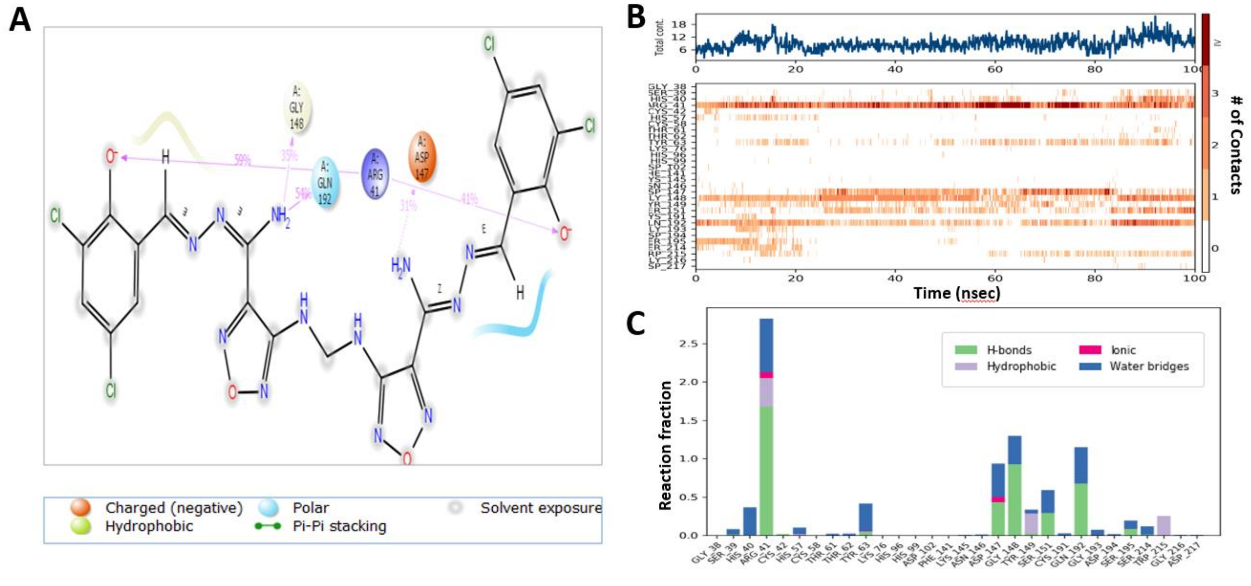

Supplementary Figure 9. Interaction diagram of M4 with TMPRSS2 protein observed during the molecular dynamic simulation. (A) Protein-ligand interaction diagram**. (B)** The top panel showed the specific contact of TMPRSS2 protein with **M4** in each trajectory course, the bottom panel showed the amino acid residues that interact with the ligand in the trajectory time frame. The residues making more than one contact were shown in darker color shade. **(C)** Schematic diagram of ligand interaction with the amino acid residues of protein during MD simulation.

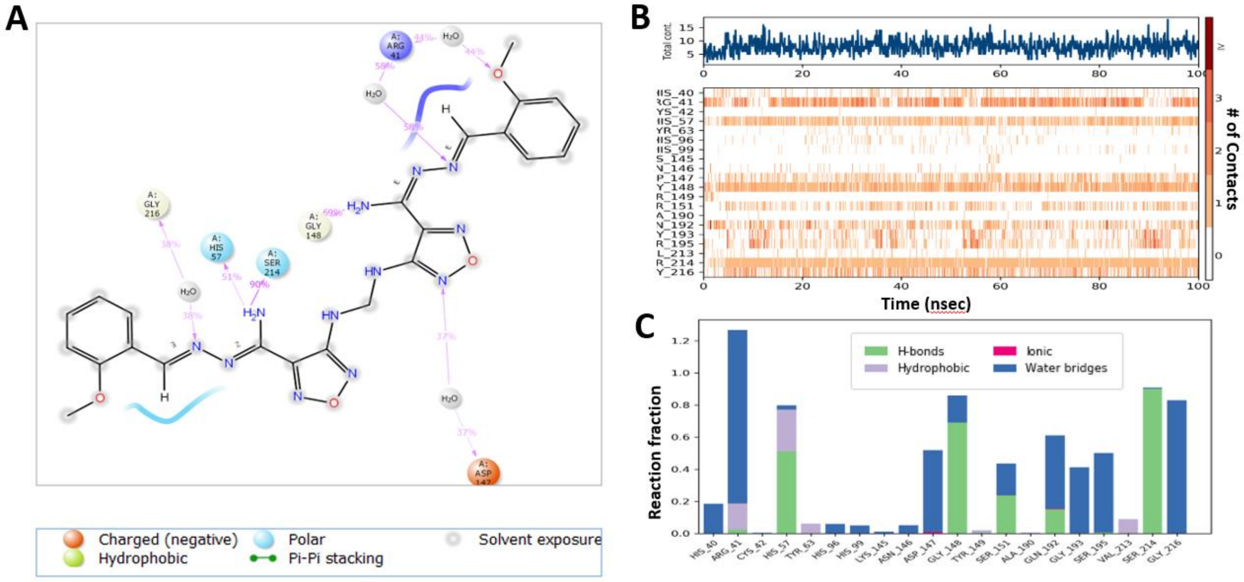

Supplementary Figure 10. Interaction diagram of M5 with TMPRSS2 protein observed during the molecular dynamic simulation. (A) Protein-ligand interaction diagram**. (B)** The top panel showed the specific contact of TMPRSS2 protein with **M5** in each trajectory course, the bottom panel showed the amino acid residues that interact with the ligand in the trajectory time frame. The residues making more than one contact were shown in darker color shade. **(C)** Schematic diagram of ligand interaction with the amino acid residues of protein during MD simulation.
